# A comparison of automatic cell identification methods for single-cell RNA sequencing data

**DOI:** 10.1186/s13059-019-1795-z

**Published:** 2019-09-09

**Authors:** Tamim Abdelaal, Lieke Michielsen, Davy Cats, Dylan Hoogduin, Hailiang Mei, Marcel J. T. Reinders, Ahmed Mahfouz

**Affiliations:** 1 Leiden Computational Biology Center, Leiden University Medical Center, Einthovenweg 20, 2333 ZC Leiden, The Netherlands; 2 Delft Bioinformatics Laboratory, Delft University of Technology, Van Mourik Broekmanweg 6, 2628 XE Delft, The Netherlands; 3 Sequencing Analysis Support Core, Department of Biomedical Data Sciences, Leiden University Medical Center, Einthovenweg 20, 2333 ZC Leiden, The Netherlands

**Keywords:** scRNA-seq, Benchmark, Classification, Cell identity

## Abstract

**Background:**

Single-cell transcriptomics is rapidly advancing our understanding of the cellular composition of complex tissues and organisms. A major limitation in most analysis pipelines is the reliance on manual annotations to determine cell identities, which are time-consuming and irreproducible. The exponential growth in the number of cells and samples has prompted the adaptation and development of supervised classification methods for automatic cell identification.

**Results:**

Here, we benchmarked 22 classification methods that automatically assign cell identities including single-cell-specific and general-purpose classifiers. The performance of the methods is evaluated using 27 publicly available single-cell RNA sequencing datasets of different sizes, technologies, species, and levels of complexity. We use 2 experimental setups to evaluate the performance of each method for within dataset predictions (intra-dataset) and across datasets (inter-dataset) based on accuracy, percentage of unclassified cells, and computation time. We further evaluate the methods’ sensitivity to the input features, number of cells per population, and their performance across different annotation levels and datasets. We find that most classifiers perform well on a variety of datasets with decreased accuracy for complex datasets with overlapping classes or deep annotations. The general-purpose support vector machine classifier has overall the best performance across the different experiments.

**Conclusions:**

We present a comprehensive evaluation of automatic cell identification methods for single-cell RNA sequencing data. All the code used for the evaluation is available on GitHub (https://github.com/tabdelaal/scRNAseq_Benchmark). Additionally, we provide a Snakemake workflow to facilitate the benchmarking and to support the extension of new methods and new datasets.

**Electronic supplementary material:**

The online version of this article (10.1186/s13059-019-1795-z) contains supplementary material, which is available to authorized users.

## Background

Single-cell RNA sequencing (scRNA-seq) provides unprecedented opportunities to identify and characterize the cellular composition of complex tissues. Rapid and continuous technological advances over the past decade have allowed scRNA-seq technologies to scale to thousands of cells per experiment [1]. A common analysis step in analyzing single-cell data involves the identification of cell populations presented in a given dataset. This task is typically solved by unsupervised clustering of cells into groups based on the similarity of their gene expression profiles, followed by cell population annotation by assigning labels to each cluster. This approach proved very valuable in identifying novel cell populations and resulted in cellular maps of entire cell lineages, organs, and even whole organisms [2–7]. However, the annotation step is cumbersome and time-consuming as it involves manual inspection of cluster-specific marker genes. Additionally, manual annotations, which are often not based on standardized ontologies of cell labels, are not reproducible across different experiments within and across research groups. These caveats become even more pronounced as the number of cells and samples increases, preventing fast and reproducible annotations.

To overcome these challenges, a growing number of classification approaches are being adapted to automatically label cells in scRNA-seq experiments. scRNA-seq classification methods predict the identity of each cell by learning these identities from annotated training data (e.g., a reference atlas). scRNA-seq classification methods are relatively new compared to the plethora of methods addressing different computational aspects of single-cell analysis (such as normalization, clustering, and trajectory inference). However, the number of classification methods is rapidly growing to address the aforementioned challenges [8, 9]. While all scRNA-seq classification methods share a common goal, i.e., accurate annotation of cells, they differ in terms of their underlying algorithms and the incorporation of prior knowledge (e.g., cell type marker gene tables).

In contrast to the extensive evaluations of clustering, differential expression, and trajectory inference methods [10–12], there is currently one single attempt comparing methods to assign cell type labels to cell clusters [13]. The lack of a comprehensive comparison of scRNA-seq classification methods leaves users without indications as to which classification method best fits their problem. More importantly, a proper assessment of the existing approaches in comparison with the baseline methods can greatly benefit new developments in the field and prevent unnecessary complexity.

Here, we benchmarked 22 classification methods to automatically assign cell identities including single-cell-specific and general-purpose classifiers. The methods were evaluated using 27 publicly available single-cell RNA sequencing datasets of different sizes, technologies, species, and complexity. The performance of the methods was evaluated based on their accuracy, percentage of unclassified cells, and computation time. We performed several experiments to cover different levels of challenge in the classification task and to test specific features or tasks such as the feature selection, scalability, and rejection experiments. We evaluated the classification performance through two experimental setups: (1) intra-dataset in which we applied 5-fold cross-validation within each dataset and (2) inter-dataset involving across datasets comparisons. The inter-dataset comparison is more realistic and more practical, where a reference dataset (e.g., atlas) is used to train a classifier which can then be applied to identify cells in new unannotated datasets. However, in order to perform well across datasets, the classifier should also perform well using the intra-dataset setup on the reference dataset. The intra-dataset experiments, albeit artificial, provide an ideal scenario to evaluate different aspects of the classification process (e.g., feature selection, scalability, and different annotation levels), regardless of the technical and biological variations across datasets. In general, most classifiers perform well across all datasets in both experimental setups (inter- and intra-dataset), including the general-purpose classifiers. In our experiments, incorporating prior knowledge in the form of marker genes does not improve the performance. We observed large variation across different methods in the computation time and classification performance in response to changing the input features and the number of cells. Our results highlight the general-purpose support vector machine (*SVM*) classifier as the best performer overall.

## Results

### Benchmarking automatic cell identification methods (intra-dataset evaluation)

We benchmarked the performance and computation time of all 22 classifiers (Table 1) across 11 datasets used for intra-dataset evaluation (Table 2). Classifiers were divided into two categories: (1) supervised methods which require a training dataset labeled with the corresponding cell populations in order to train the classifier or (2) prior-knowledge methods, for which either a marker gene file is required as an input or a pretrained classifier for specific cell populations is provided.

**Table 1:** Automatic cell identification methods included in this study

Name	Version	Language	Underlying classifier	Prior knowledge	Rejection option	Reference
Garnett	0.1.4	R	Generalized linear model	Yes	Yes	[14]
Moana	0.1.1	Python	SVM with linear kernel	Yes	No	[15]
DigitalCellSorter	GitHub version: e369a34	Python	Voting based on cell type markers	Yes	No	[16]
SCINA	1.1.0	R	Bimodal distribution fitting for marker genes	Yes	No	[17]
scVI	0.3.0	Python	Neural network	No	No	[18]
Cell-BLAST	0.1.2	Python	Cell-to-cell similarity	No	Yes	[19]
ACTINN	GitHub version: 563bcc1	Python	Neural network	No	No	[20]
LAmbDA	GitHub version: 3891d72	Python	Random forest	No	No	[21]
scmapcluster	1.5.1	R	Nearest median classifier	No	Yes	[22]
scmapcell	1.5.1	R	kNN	No	Yes	[22]
scPred	0.0.0.9000	R	SVM with radial kernel	No	Yes	[23]
CHETAH	0.99.5	R	Correlation to training set	No	Yes	[24]
CaSTLe	GitHub version: 258b278	R	Random forest	No	No	[25]
SingleR	0.2.2	R	Correlation to training set	No	No	[26]
scID	0.0.0.9000	R	LDA	No	Yes	[27]
singleCellNet	0.1.0	R	Random forest	No	No	[28]
LDA	0.19.2	Python	LDA	No	No	[29]
NMC	0.19.2	Python	NMC	No	No	[29]
RF	0.19.2	Python	RF (50 trees)	No	No	[29]
SVM	0.19.2	Python	SVM (linear kernel)	No	No	[29]
SVM_rejection_	0.19.2	Python	SVM (linear kernel)	No	Yes	[29]
kNN	0.19.2	Python	kNN (*k* = 9)	No	No	[29]

**Table 2:** Overview of the datasets used during this study

Dataset	No. of cells	No. of genes	No. of cell populations (> 10 cells)	Description	Protocol	Reference
Baron (Mouse)^a^	1886	14,861	13 (9)	Mouse pancreas	inDrop	[30]
Baron (Human)^a,b^	8569	17,499	14 (13)	Human pancreas	inDrop	[30]
Muraro^a,b^	2122	18,915	9 (8)	Human pancreas	CEL-Seq2	[31]
Segerstolpe^a,b^	2133	22,757	13 (9)	Human pancreas	SMART-Seq2	[32]
Xin^a,b^	1449	33,889	4 (4)	Human pancreas	SMARTer	[33]
CellBench 10X^a,b^	3803	11,778	5 (5)	Mixture of five human lung cancer cell lines	10X chromium	[34]
CellBench CEL-Seq2^a,b^	570	12,627	5 (5)	Mixture of five human lung cancer cell lines	CEL-Seq2	[34]
TM^a^	54,865	19,791	55 (55)	Whole *Mus musculus*	SMART-Seq2	[6]
AMB^a^	12,832	42,625	4/22/110 (3/16/92)	Primary mouse visual cortex	SMART-Seq v4	[35]
Zheng sorted^a^	20,000	21,952	10 (10)	FACS-sorted PBMC	10X CHROMIUM	[36]
Zheng 68K^a^	65,943	20,387	11 (11)	PBMC	10X CHROMIUM	[36]
VISp^b^ (Mouse)	12,832	42,625	3/36 (3/34)	Primary visual cortex	SMART-Seq v4	[35]
ALM^b^ (Mouse)	8758	42,461	3/37 (3/34)	Anterior lateral motor area	SMART-Seq v4	[35]
MTG^b^ (Human)	14,636	16,161	3/35 (3/34)	Middle temporal gyrus	SMART-Seq v4	[37]
PbmcBench pbmc1.10Xv2^b^	6444	33,694	9 (9)	PBMC	10X version 2	[38]
PbmcBench pbmc1.10Xv3^b^	3222	33,694	8 (8)	PBMC	10X version 3	[38]
PbmcBench pbmc1.CL^b^	253	33,694	7 (7)	PBMC	CEL-Seq2	[38]
PbmcBench pbmc1.DR^b^	3222	33,694	9 (9)	PBMC	Drop-Seq	[38]
PbmcBench pbmc1.iD^b^	3222	33,694	7 (7)	PBMC	inDrop	[38]
PbmcBench pbmc1.SM2^b^	253	33,694	6 (6)	PBMC	SMART-Seq2	[38]
PbmcBench pbmc1.SW^b^	3176	33,694	7 (7)	PBMC	Seq-Well	[38]
PbmcBench pbmc2.10Xv2^,b^	3362	33,694	9 (9)	PBMC	10X version 2	[38]
PbmcBench pbmc2.CL^b^	273	33,694	5 (5)	PBMC	CEL-Seq2	[38]
PbmcBench pbmc2.DR^b^	3362	33,694	6 (6)	PBMC	Drop-Seq	[38]
PbmcBench pbmc2.iD^b^	3362	33,694	9 (9)	PBMC	inDrop	[38]
PbmcBench pbmc2.SM2^b^	273	33,694	6 (6)	PBMC	SMART-Seq2	[38]
PbmcBench pbmc2.SW^b^	551	33,694	4 (4)	PBMC	Seq-Well	[38]

^a^Used for intra-dataset evaluation

^b^Used for inter-dataset evaluation

The datasets used in this study vary in the number of cells, genes, and cell populations (annotation level), in order to represent different levels of challenges in the classification task and to evaluate how each classifier performs in each case (Table 2). They include relatively typical sized scRNA-seq datasets (1500–8500 cells), such as the 5 pancreatic datasets (Baron Mouse, Baron Human, Muraro, Segerstolpe, and Xin), which include both mouse and human pancreatic cells and vary in the sequencing protocol used. The Allen Mouse Brain (AMB) dataset is used to evaluate how the classification performance changes when dealing with different levels of cell population annotation as the AMB dataset contains three levels of annotations for each cell (3, 16, or 92 cell populations), denoted as AMB3, AMB16, and AMB92, respectively. The Tabula Muris (TM) and Zheng 68K datasets represent relatively large scRNA-seq datasets (> 50,000 cells) and are used to assess how well the classifiers scale with large datasets. For all previous datasets, cell populations were obtained through clustering. To assess how the classifiers perform when dealing with sorted populations, we included the CellBench dataset and the Zheng sorted dataset, representing sorted populations for lung cancer cell lines and peripheral blood mononuclear cells (PBMC), respectively. Including the Zheng sorted and Zheng 68K datasets allows the benchmarking of 4 prior-knowledge classifiers, since the marker gene files or pretrained classifiers are available for the 4 classifiers for PBMCs.

### All classifiers perform well in intra-dataset experiments

**Fig. 1:**
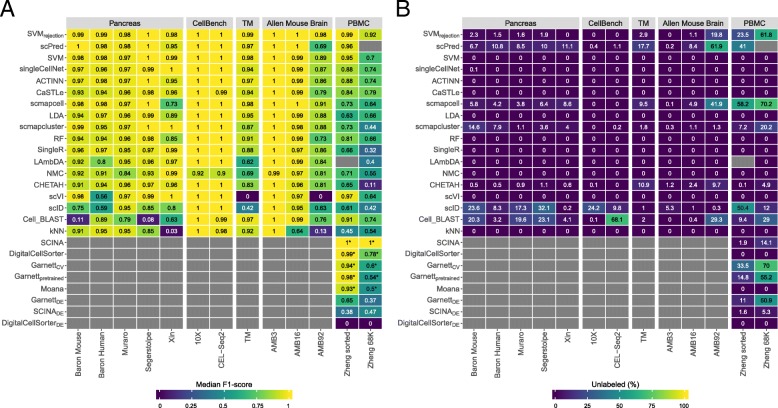
Performance comparison of supervised classifiers for cell identification using different scRNA-seq datasets. Heatmap of the **a** median F1-scores and **b** percentage of unlabeled cells across all cell populations per classifier (rows) per dataset (columns). Gray boxes indicate that the corresponding method could not be tested on the corresponding dataset. Classifiers are ordered based on the mean of the median F1-scores. Asterisk (*) indicates that the prior-knowledge classifiers, *SCINA*, *DigitalCellSorter*, *Garnett*_*CV*_, *Garnett*_*pretrained*_, and *Moana*, could not be tested on all cell populations of the PBMC datasets. *SCINA*_*DE*_, *Garnett*_*DE*_, and *DigitalCellSorter*_*DE*_ are versions of *SCINA*, *Garnett*_*CV*_, and *DigitalCellSorter*; the marker genes are defined using differential expression from the training data. Different numbers of marker genes, 5, 10, 15, and 20, were tested, and the best result is shown here. *SCINA*, *Garnett*, and *DigitalCellSorter* produced the best result for the Zheng sorted dataset using 20, 15, and 5 markers, and for the Zheng 68K dataset using 10, 5, and 5 markers, respectively

Generally, all classifiers perform well in the intra-dataset experiments, including the general-purpose classifiers (Fig. 1). However, *Cell-BLAST* performs poorly for the Baron Mouse and Segerstople pancreatic datasets. Further, *scVI* has low performance on the deeply annotated datasets TM (55 cell populations) and AMB92 (92 cell populations), and *kNN* produces low performance for the Xin and AMB92 datasets.

For the pancreatic datasets, the best-performing classifiers are *SVM*, *SVM*_*rejection*_, *scPred*, *scmapcell*, *scmapcluster*, *scVI*, *ACTINN*, *singleCellNet*, *LDA*, and *NMC*. *SVM* is the only classifier to be in the top five list for all five pancreatic datasets, while *NMC*, for example, appears only in the top five list for the Xin dataset. The Xin dataset contains only four pancreatic cell types (alpha, beta, delta, and gamma) making the classification task relatively easy for all classifiers, including *NMC*. Considering the median F1-score alone to judge the classification performance can be misleading since some classifiers incorporate a rejection option (e.g., *SVM*_*rejection*_, *scmapcell*, *scPred*), by which a cell is assigned as “unlabeled” if the classifier is not confident enough. For example, for the Baron Human dataset, the median F1-score for *SVM*_*rejection*_, *scmapcell*, *scPred*, and *SVM* is 0.991, 0.984, 0.981, and 0.980, respectively (Fig. 1a). However, *SVM*_*rejection*_, *scmapcell*, and *scPred* assigned 1.5%, 4.2%, and 10.8% of the cells, respectively, as unlabeled while *SVM* (without rejection) classified 100% of the cells with a median F1-score of 0.98 (Fig. 1b). This shows an overall better performance for *SVM* and *SVM*_*rejection*_, with higher performance and less unlabeled cells.

The CellBench 10X and CEL-Seq2 datasets represent an easy classification task, where the five sorted lung cancer cell lines are quite separable [34]. All classifiers have an almost perfect performance on both CellBench datasets (median F1-score ≈ 1).

For the TM dataset, the top five performing classifiers are *SVM*_*rejection*_, *SVM*, *scmapcell*, *Cell-BLAST*, and *scPred* with a median F1-score > 0.96, showing that these classifiers can perform well and scale to large scRNA-seq datasets with a deep level of annotation. Furthermore, *scmapcell* and *scPred* assigned 9.5% and 17.7% of the cells, respectively, as unlabeled, which shows a superior performance for *SVM*_*rejection*_ and *SVM*, with a higher median F1-score and 2.9% and 0% unlabeled cells, respectively.

### Performance evaluation across different annotation levels

We used the AMB dataset with its three different levels of annotations, to evaluate the classifiers’ performance behavior with an increasing number of smaller cell populations within the same dataset. For AMB3, the classification task is relatively easy, differentiating between three major brain cell types (inhibitory neurons, esxcitatory neurons, and non-neuronal). All classifiers perform almost perfectly with a median F1-score > 0.99 (Fig. 1a). For AMB16, the classification task becomes slightly more challenging and the performance of some classifiers drops, especially *kNN*. The top five classifiers are *SVM*_*rejection*_, *scmapcell*, *scPred*, *SVM*, and *ACTINN*, where *SVM*_*rejection*_, *scmapcell*, and *scPred* assigned 1.1%, 4.9%, and 8.4% of the cells as unlabeled, respectively. For the deeply annotated AMB92 dataset, the performance of all classifiers drops further, specially for *kNN* and *scVI*, where the median F1-score is 0.130 and zero, respectively. The top five classifiers are *SVM*_*rejection*_, *scmapcell*, *SVM*, *LDA*, and *scmapcluster*, with *SVM*_*rejection*_ assigning less cells as unlabeled compared to *scmapcell* (19.8% vs 41.9%), and once more, *SVM*_*rejection*_ shows improved performance over *scmapcell* (median F1-score of 0.981 vs 0.906). These results show an overall superior performance for general-purpose classifiers (*SVM*_*rejection*_, *SVM*, and *LDA*) compared to other scRNA-seq-specific classifiers across different levels of cell population annotation.

Instead of only looking at the median F1-score, we also evaluated the F1-score per cell population for each classifier (Additional file 1: Figure S1). We confirmed previous conclusions that *kNN* performance drops with deep annotations which include smaller cell populations (Additional file 1: Figure S1B-C), and *scVI* poorly performs on the deeply annotated AMB92 dataset. Additionally, we observed that some cell populations are much harder to classify compared to other populations. For example, most classifiers had a low performance on the *Serpinf1* cells in the AMB16 dataset.

### Incorporating prior-knowledge does not improve intra-dataset performance on PBMC data

For the two PBMC datasets (Zheng 68K and Zheng sorted), the prior-knowledge classifiers *Garnett*, *Moana*, *DigitalCellSorter*, and *SCINA* could be evaluated and benchmarked with the rest of the classifiers. Although the best-performing classifier on Zheng 68K is *SCINA* with a median F1-score of 0.998, this performance is based only on 3, out of 11, cell populations (Monocytes, B cells, and NK cells) for which marker genes are provided. Additional file 1: Table S1 summarizes which PBMC cell populations can be classified by the prior-knowledge methods. Interestingly, none of the prior-knowledge methods showed superior performance compared to other classifiers, despite the advantage these classifiers have over other classifiers given they are tested on fewer cell populations due to the limited availability of marker genes. *Garnett*, *Moana*, and *DigitalCellSorter* could be tested on 7, 7, and 5 cell populations, respectively (Additional file 1: Table S1). Besides *SCINA*, the top classifiers for the Zheng 68K dataset are *CaSTLe*, *ACTINN*, *singleCellNet*, and *SVM*. *SVM*_*rejection*_ and *Cell-BLAST* show high performance, at the expense of a high rejection rate of 61.8% and 29%, respectively (Fig. 1). Moreover, *scPred* failed when tested on the Zheng 68K dataset. Generally, all classifiers show relatively lower performance on the Zheng 68K dataset compared to other datasets, as the Zheng 68K dataset contains 11 immune cell populations which are harder to differentiate, particularly the T cell compartment (6 out of 11 cell populations). This difficulty of separating these populations was previously noted in the original study [36]. Also, the confusion matrices for *CaSTLe*, *ACTINN*, *singleCellNet*, and *SVM* clearly indicate the high similarity between cell populations, such as (1) monocytes with dendritic cells, (2) the 2 CD8+ T populations, and (3) the 4 CD4+ T populations (Additional file 1: Figure S2).

The classification of the Zheng sorted dataset is relatively easier compared to the Zheng 68K dataset, as almost all classifiers show improved performance (Fig. 1), with the exception that *LAmbDA* failed while being tested on the Zheng sorted dataset. The prior-knowledge methods show high performance (median F1-score > 0.93), which is still comparable to other classifiers such as *SVM*_*rejection*_, *scVI*, *scPred*, and *SVM*. Yet, the supervised classifiers do not require any marker genes, and they can predict more (all) cell populations.

### The performance of prior-knowledge classifiers strongly depends on the selected marker genes

Some prior-knowledge classifiers, *SCINA*, *DigitalCellSorter*, and *Garnett*_*CV*_, used marker genes to classify the cells. For the PBMC datasets, the number of marker genes per cell population varies across classifiers (2–161 markers) and the marker genes show very little overlap. Only one B cell marker gene, CD79A, is shared by all classifiers while none of the marker genes for the other cell populations is shared by the three classifiers. We analyzed the effect of the number of marker genes, mean expression, dropout rate, and the specificity of each marker gene (beta score, see the “Methods” section) on the performance of the classifier (Additional file 1: Figure S3). The dropout rate and marker specificity (beta-score) are strongly correlated with the median F1-score, highlighting that the performance does not only depend on biological knowledge, but also on technical factors.

The difference between the marker genes used by each method underscores the challenge of marker gene selection, especially for smaller cell populations. Moreover, public databases of cell type markers (e.g., PanglaoDB [39] and CellMarker [40]) often provide different markers for the same population. For example, CellMarker provides 33 marker genes for B cells, while PanglaoDB provides 110 markers, with only 11 marker genes overlap between the two databases.

Given the differences between “expert-defined” markers and the correlation of classification performance and technical dataset-specific features (e.g., dropout rate), we tested if the performance of prior-knowledge methods can be improved by automatically selecting marker genes based on differential expression. Through the cross-validation scheme, we used the training folds to select the marker genes of each cell population based on differential expression (see the “Methods” section) and later used these markers to evaluate the classifiers’ performance on the testing fold. We tested this approach on the two PBMC datasets, Zheng sorted and Zheng 68K for different numbers of marker genes (5, 10, 15, and 20 markers). In Fig. 1, the best result across the number of markers for *SCINA*_*DE*_, *Garnett*_*DE*_, and *DigitalCellSorter*_*DE*_ are shown.

The median F1-score obtained using the differential expression-defined markers is significantly lower compared to the original versions of classifiers using the markers defined by the authors. This lower performance is in part due to the low performance on challenging populations, such as subpopulations of CD4+ and CD8+ T cell populations (F1-score ≤ 0.68) (Additional file 1: Figure S4). These challenging populations are not identified by the original classifiers since the markers provided by the authors only considered annotations at a higher level (Additional file 1: Table S1). For example, the median F1-score of _*SCINADE*_ on Zheng sorted is 0.38, compared to a median F1-score of 1.0 for *SCINA* (using the original markers defined by the authors). However, *SCINA* only considers three cell populations: CD14+ monocytes, CD56+ NK cells, and CD19+ B cells. If we only consider these cell populations for *SCINA*_*DE*_, this results in a median F1-score of 0.95.

We observed that the optimal number of marker genes varies per classifier and dataset. For the Zheng sorted dataset, the optimal number of markers is 5, 15, and 20 for *DigitalCellSorter*_*DE*_, *Garnett*_*DE*_, and *SCINA*_*DE*_, respectively, while for Zheng 68K, this is 5, 5, and 10. All together, these results illustrate the dependence of the classification performance on the careful selection of marker genes which is evidently a challenging task.

### Classification performance depends on dataset complexity

**Fig. 2:**
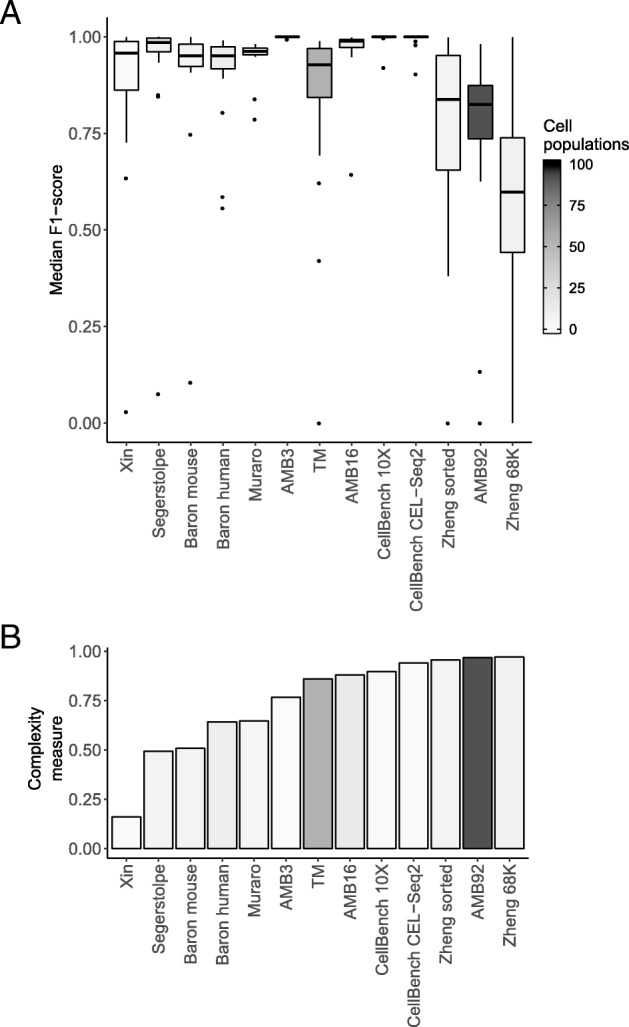
Complexity of the datasets compared to the performance of the classifiers. **a** Boxplots of the median F1-scores of all classifiers for each dataset used during the intra-dataset evaluation. **b** Barplots describing the complexity of the datasets (see the “Methods” section). Datasets are ordered based on complexity. Box- and bar plots are colored according to the number of cell populations in each dataset

A major aspect affecting the classification performance is the complexity of the dataset at hand. We described the complexity of each dataset in terms of the pairwise similarity between cell populations (see the “Methods” section) and compared the complexity to the performance of the classifiers and the number of cell populations in a dataset (Fig. 2). When the complexity and/or the number of cell populations of the dataset increases, the performance generally decreases. The performance of all classifiers is relatively low on the Zheng 68K dataset, which can be explained by the high pairwise correlations between the mean expression profiles of each cell population (Additional file 1: Figure S5). These correlations are significantly lower for the TM and AMB92 datasets, justifying the higher performance of the classifiers on these two datasets (Additional file 1: Figures S6–S7). While both TM and AMB92 have more cell populations (55 and 92, respectively) compared to Zheng 68K (11 populations), these populations are less correlated to one another, making the task easier for all the classifiers.

### Performance evaluation across datasets (inter-dataset evaluation)

While evaluating the classification performance within a dataset (intra-dataset) is important, the realistic scenario in which a classifier is useful requires cross-dataset (i.e., inter-dataset) classification. We used 22 datasets (Table 2) to test the classifiers’ ability to predict cell identities in a dataset that was not used for training. First, we tested the classifiers’ performance across different sequencing protocols, applied to the same samples within the same lab using the two CellBench datasets. We evaluated the classification performance when training on one protocol and testing on the other. Similar to the intra-dataset evaluation result, all classifiers performed well in this case (Additional file 1: Figure S8).

**Fig. 3:**
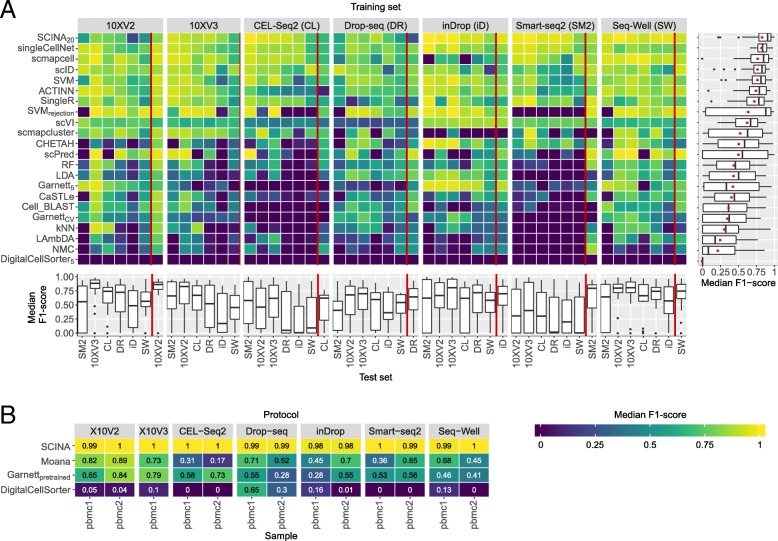
Classification performance across the PbmcBench datasets. **a** Heatmap showing the median F1-scores of the supervised classifiers for all train-test pairwise combination across different protocols. The training set is indicated in the gray box on top of the heatmap, and the test set is indicated using the column labels below. Results shown to the left of the red line represent the comparison between different protocols using sample pbmc1. Results shown to the right of the red line represent the comparison between different samples using the same protocol, with pbmc 1 used for training and pbmc2 used for testing. Boxplots on the right side of the heatmap summarize the performance of each classifier across all experiments. The mean of the median F1-scores, also used to order the classifiers, is indicated in the boxplots using a red dot. Boxplots underneath the heatmap summarize the performance of the classifiers per experiment. For *SCINA*_*DE*_, *Garnett*_*DE*_, and *DigitalCellSorter*_*DE*_, different numbers of marker genes were tested. Only the best result is shown here. **b** Median F1-score of the prior-knowledge classifiers on both samples of the different protocols. The protocol is indicated in the gray box on top of the heatmap, and the sample is indicated with the labels below. Classifiers are ordered based on their mean performance across all datasets

Second, we tested the classification performance on the PbmcBench datasets, which represent a more extensive protocol comparison. PbmcBench consists of 2 samples (pbmc1 and pbmc2), sequenced using 7 different protocols (Table 2) with the exception that 10Xv3 was not applied to the pbmc2 sample. We used the pbmc1 datasets to evaluate the classification performance of all pairwise train-test combinations between the 7 protocols (42 experiments, see the “Methods” section). Moreover, we extended the evaluation to include comparisons across different samples for the same protocol, using pbmc1 and pbmc2 (6 experiments, see the “Methods” section). All 48 experiment results are summarized in Fig. 3. Overall, several classifiers performed well including *SCINA*_*DE*_ using 20 marker genes, *singleCellNet*, *scmapcell*, *scID*, and *SVM*, with an average median F1-score > 0.75 across all 48 experiments (Fig. 3a, Additional file 1: Figure S9A). *SCINA*_*DE*_, *Garnett*_*DE*_, and *DigitalCellSorter*_*DE*_ were tested using 5, 10, 15, and 20 marker genes; Fig. 3a shows the best result for each classifier, where *SCINA*_*DE*_ and *Garnett*_*DE*_ performed best using 20 and 5 marker genes, respectively, while *DigitalCellSorter*_*DE*_ had a median F1-score of 0 during all experiments using all different numbers of marker genes. *DigitalCellSorter*_*DE*_ could only identify B cells in the test sets, usually with an F1-score between 0.8 and 1.0, while the F1-score for all other cell populations was 0.

We also tested the prior-knowledge classifiers on all 13 PbmcBench datasets. The prior-knowledge classifiers showed lower performance compared to other classifiers (average median F1-score < 0.6), with the exception of *SCINA* which was only tested on three cell populations (Fig. 3b, Additional file 1: Figure S9B). These results are in line with our previous conclusions from the Zheng sorted and Zheng 68K datasets in the intra-dataset evaluation.

Comparing the performance of the classifiers across the different protocols, we observed a higher performance for all classifiers for specific pairs of protocols. For example, all classifiers performed well when trained on 10Xv2 and tested on 10Xv3, and vice versa. On the other hand, other pairs of protocols had a good performance only in one direction, training on Seq-Well produced good predictions on 10Xv3, but not the other way around. Compared to all other protocols, the performance of all classifiers was low when they were either trained or tested on Smart-seq2 data. This can, in part, be due to the fact that Smart-seq2 data does not contain unique molecular identifier (UMI), in contrast to all other protocols.

**Fig. 4:**
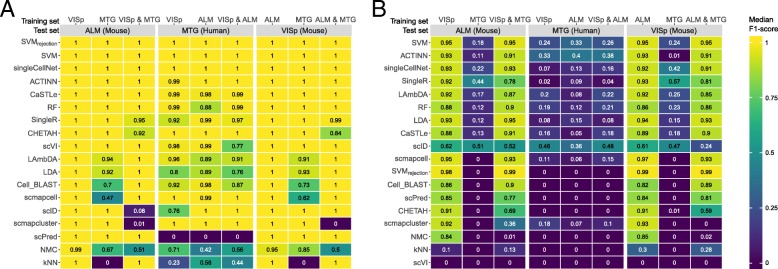
Classification performance across brain datasets. Heatmaps show the median F1-scores of the supervised classifiers when tested on **a** major lineage annotation with three cell populations and **b** deeper level of annotation with 34 cell populations. The training sets are indicated using the column labels on top of the heatmap. The test set is indicated in the gray box. In each heatmap, the classifiers are ordered based on their mean performance across all experiments

We also tested the classification performance using the 3 brain datasets, VISp, ALM, and MTG (Table 2), which allowed us to compare the performances across species (mouse and human) as well as single-cell RNA-seq (used in VISp and ALM) vs single-nucleus RNA-seq (used in MTG). We tested all possible train-test combinations for both levels of annotation, three major brain cell types (inhibitory neurons, excitatory neurons, and non-neuronal cells), and the deeper annotation level with 34 cell populations (18 experiments, see the “Methods” section). Prediction of the three major cell types was easy, where almost all classifiers showed high performance (Fig. 4a) with some exceptions. For example, *scPred* failed the classification task completely when testing on the MTG dataset, producing 100% unlabeled cells (Additional file 1: Figure S10A). Predicting the 34 cell populations turned out to be a more challenging task, especially when the MTG human dataset is included either as training or testing data, resulting in significantly lower performance across all classifiers (Fig. 4b). Across all nine experiments at the deeper annotation, the top-performing classifiers were *SVM*, *ACTINN*, *singleCellNet*, *SingleR*, and *LAmbDA*, with almost 0% unlabeled cells (Additional file 1: Figure S10B).

Finally, to evaluate the classification performance across different protocols and different labs, we used the four human pancreatic datasets: Baron Human, Muraro, Segerstople, and Xin (see the “Methods” section, Additional file 1: Table S2). We tested four combinations by training on three datasets and test on one dataset, in which case the classification performance can be affected by batch differences between the datasets. We evaluated the performance of the classifiers when trained using the original data as well as aligned data using the mutual nearest neighbor (MNN) method [41]. Additional file 1: Figure S11 shows UMAPs [42] of the combined dataset before and after alignment, demonstrating better grouping of pancreatic cell types after alignment.

**Fig. 5:**
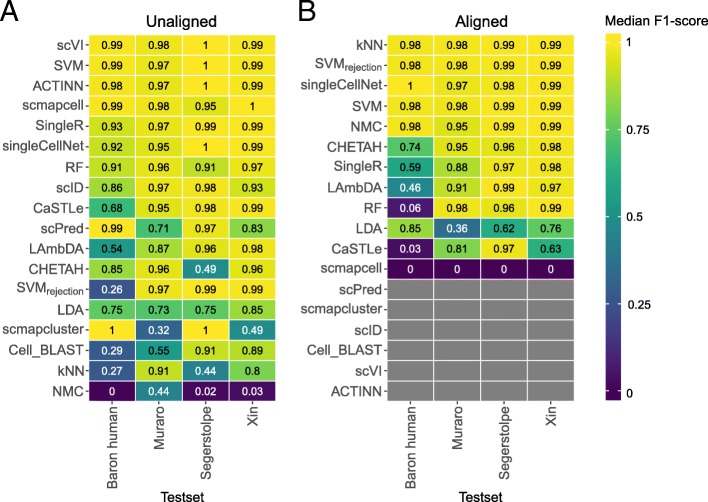
Classification performance across pancreatic datasets. Heatmaps showing the median F1-score for each classifier for the **a** unaligned and **b** aligned datasets. The column labels indicate which of the four datasets was used as a test set, in which case the other three datasets were used as training. Gray boxes indicate that the corresponding method could not be tested on the corresponding dataset. In each heatmap, the classifiers are ordered based on their mean performance across all experiments

For the original (unaligned) data, the best-performing classifiers across all four experiments are *scVI*, *SVM*, *ACTINN*, *scmapcell*, and *SingleR* (Fig. 5a, Additional file 1: Figure S12A). For the aligned data, the best-performing classifiers are *kNN*, *SVM*_*rejection*_, *singleCellNet*, *SVM*, and *NMC* (Fig. 5b, Additional file 1: Figure S12B). Some classifiers benefit from aligning datasets such as *SVM*_*rejection*_, *kNN*, *NMC*, and *singleCellNet*, resulting in higher median F1-scores (Fig. 5). On the other hand, some other classifiers failed the classification task completely, such as *scmapcell* which labels all cells as unlabeled. Some other classifiers failed to run over the aligned datasets, such as *ACTINN*, *scVI*, *Cell-BLAST*, *scID*, *scmapcluster*, and *scPred*. These classifiers work only with positive gene expression data, while the aligned datasets contain positive and negative gene expression values.

### Rejection option evaluation

Classifiers developed for scRNA-seq data often incorporate a rejection option to identify cell populations in the test set that were not seen during training. These populations cannot be predicted correctly and therefore should remain unassigned. To test whether the classifiers indeed leave these unseen populations unlabeled, we applied two different experiments using negative controls of different tissues and using unseen populations of the same tissue.

**Fig. 6:**
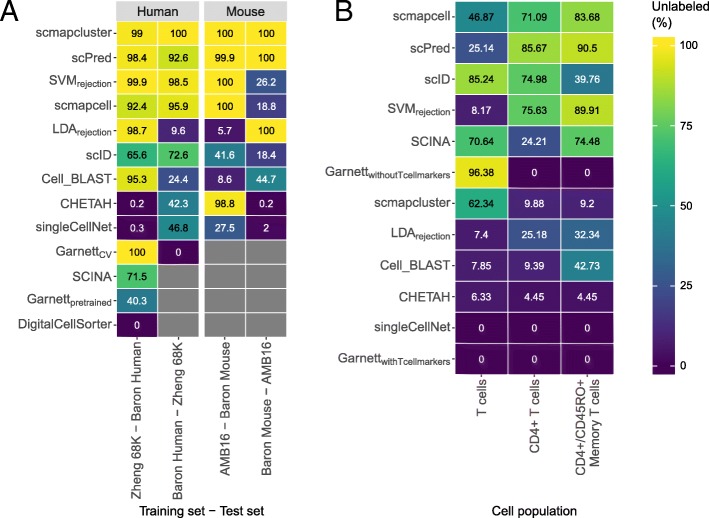
Performance of the classifiers during the rejection experiments. **a** Percentage of unlabeled cells during the negative control experiment for all the classifiers with a rejection option. The prior-knowledge classifiers could not be tested on all datasets, and this is indicated with a gray box. The species of the dataset is indicated in the gray box on top. Column labels indicate which datasets are used for training and testing. **b** Percentage of unlabeled cells for all classifiers with a rejection option when a cell population was removed from the training set. Column labels indicate which cell population was removed. This cell population was used as a test set. In both **a** and **b**, the classifiers are sorted based on their mean performance across all experiments

First, the classifiers were trained on a data set from one tissue (e.g., pancreas) and used to predict cell populations of a completely different tissue (e.g., brain) [22]. The methods should thus reject all (100%) of the cells in the test dataset. We carried out four different negative control experiments (see the “Methods” section, Fig. 6a). *scmapcluster* and *scPred* have an almost perfect score for all four combinations, rejecting close 100% of the cells. Other top-performing methods for this task, *SVM*_*rejection*_ and *scmapcell*, failed when trained on mouse pancreatic data and tested on mouse brain data. All labeled cells of the AMB16 dataset are predicted to be beta cells in this case. The prior-knowledge classifiers, *SCINA*, *Garnett*_*pretrained*_, and *DigitalCellSorter*, could only be tested on the Baron Human pancreatic dataset. *Garnett*_*CV*_ could, on top of that, also be trained on the Baron Human dataset and tested on the Zheng 68K dataset. During the training phase, *Garnett*_*CV*_ tries to find representative cells for the cell populations described in the marker gene file. Being trained on Baron Human using the PBMC marker gene file, it should not be able to find any representatives, and therefore, all cells in the Zheng 68K dataset should be unassigned. Surprisingly, *Garnett*_*CV*_ still finds representatives for PBMC cells in the pancreatic data, and thus, the cells in the test set are labeled. However, being trained on the PBMC dataset and tested on the pancreatic dataset, it does have a perfect performance.

To test the rejection option in a more realistic and challenging scenario, we trained the classifiers on some cell populations from one dataset and used the held out cell populations in the test set (see the “Methods” section). Since the cell populations in the test set were not seen during training, they should remain unlabeled. Here, the difficulty of the task was gradually increased (Additional file 1: Table S3). First, all the T cells were removed from the training set. Next, only the CD4+ T cells were removed. Finally, only CD4+/CD45RO+ memory T cells, a subpopulation of the CD4+ T cells, were removed. The top-performing methods for this task are *scmapcell*, *scPred*, *scID*, *SVM*_*rejection*_, and *SCINA* (Fig. 6b)*.* We expected that rejecting T cells would be a relatively easy task as they are quite distinct from all other cell populations in the dataset. It should thus be comparable to the negative control experiment. Rejecting CD4+/CD45RO+ memory T cells, on the other hand, would be more difficult as they could easily be confused with all other subpopulations of CD4+ T cells. Surprisingly, almost all classifiers, except for *scID* and *scmapcluster*, show the opposite.

To better understand this unexpected performance, we analyzed the labels assigned by *SVM*_*rejection*_. In the first task (T cells removed from the training set), *SVM*_*rejection*_ labels almost all T cells as B cells. This can be explained by the fact that *SVM*_*rejection*_, and most classifiers for that matter, relies on the classification posterior probabilities to assign labels but ignores the actual similarity between each cell and the assigned population. In task 2 (CD4+ T cells were removed), there were two subpopulations of CD8+ T cells in the training set. In that case, two cell populations are equally similar to the cells in the test set, resulting in low posterior probabilities for both classes and thus the cells in the test set remain unlabeled. If one of these CD8+ T cell populations was removed from the training set, only 10.53% instead of 75.57% of the CD4+ T cells were assigned as unlabeled by *SVM*_*rejection*_. All together, our results indicate that despite the importance of incorporating a rejection option in cell identity classifiers, the implementation of this rejection option remains challenging.

### Performance sensitivity to the input features

During the intra-datasets cross-validation experiment described earlier, we used all features (genes) as input to the classifiers. However, some classifiers suffer from overtraining when too many features are used. Therefore, we tested the effect of feature selection on the performance of the classifiers. While different strategies for feature selection in scRNA-seq classification experiments exist, selecting genes with a higher number of dropouts compared to the expected number of dropouts has been shown to outperform other methods [22, 43]. We selected subsets of features from the TM dataset using the dropout method. In the experiments, we used the top 100, 200, 500, 1000, 2000, 5000, and 19,791 (all) genes. Some classifiers include a built-in feature selection method which is used by default. To ensure that all methods use the same set of features, the built-in feature selection was turned off during these experiments.

**Fig. 7:**
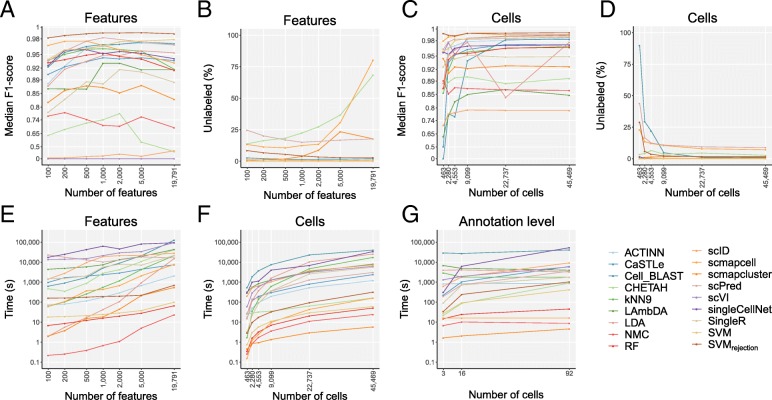
Computation time evaluation across different numbers of features, cells, and annotation levels. Line plots show **a** the median F1-score, **b** percentage of unlabeled cells, and **e** computation time of each classifier applied to the TM dataset with the top 100, 200, 500, 1000, 2000, 5000, and 19,791 (all) genes as input feature sets. Genes were ranked based on dropout-based feature selection. **c** The median F1-score, **d** percentage of unlabeled cells, and **f** computation time of each classifier applied to the downsampled TM datasets containing 463, 2280, 4553, 9099, 22,737, and 45,469 (all) cells. **g** The computation time of each classifier is plotted against the number of cell populations. Note that the *y*-axis is 100^x scaled in **a** and **c** and log-scaled in **e**–**g**. The *x*-axis is log-scaled in **a**–**f**

Some methods are clearly overtrained when the number of features increases (Fig. 7a). For example, *scmapcell* shows the highest median F1-score when using less features, and the performance drops when the number of features increases. On the other hand, the performance of other classifiers, such as *SVM*, keeps improving when the number of features increases. These results indicate that the optimal number of features is different for each classifier.

Looking at the median F1-score, there are several methods with a high maximal performance. *Cell-BLAST*, *ACTINN*, *scmapcell*, *scPred*, *SVM*_*rejection*_, and *SVM* all have a median F1-score higher than 0.97 for one or more of the feature sets. Some of these well-performing methods, however, leave many cells unlabeled. *scmapcell* and *scPred*, for instance, yield a maximum median F1-score of 0.976 and 0.982, respectively, but 10.7% and 15.1% of the cells are assigned as unlabeled (Fig. 7b). On the other hand, *SVM*_*rejection*_ has the highest median F1-score (0.991) overall with only 2.9% unlabeled. Of the top-performing classifiers, only *ACTINN* and *SVM* label all the cells. Overall *SVM* shows the third highest performance with a score of 0.979.

### Scalability: performance sensitivity to the number of cells

scRNA-seq datasets vary significantly across studies in terms of the number of cells analyzed. To test the influence of the size of the dataset on the performance of the classifier, we downsampled the TM dataset in a stratified way (i.e., preserving population frequencies) to 1, 5, 10, 20, 50, and 100% of the original number of 45,469 cells (see the “Methods” section) and compared the performance of the classifiers (Fig. 7c, d). Using less than 500 cells in the dataset, most classifiers have a relatively high performance. Only *scID*, *LAmbDA*, *CaSTLe*, and *Cell-BLAST* have a median F1-score below 0.85. Surprisingly, *SVM*_*rejection*_ has almost the same median F1-score when using 1% of the data as when using all data (0.993 and 0.994). It must be noted here, however, that the percentage of unlabeled cells decreases significantly (from 28.9% to 1.3%). Overall, the performance of all classifiers stabilized when tested on ≥ 20% (9099 cells) of the original data.

### Running time evaluation

To compare the runtimes of the classification methods and see how they scale when the number of cells increases, we compared the number of cells in each dataset with the computation time of the classifiers (Additional file 1: Figure S13). Overall, big differences in the computation time can be observed when comparing the different methods. *SingleR* showed the highest computation time overall. Running *SingleR* on the Zheng 68K dataset took more than 39 h, while *scmapcluster* was finished within 10 s on this dataset. Some of the methods have a high runtime for the small datasets. On the smallest dataset, Xin, all classifiers have a computation time < 5 min, with most classifiers finishing within 60 s. *Cell-BLAST*, however, takes more than 75 min. In general, all methods show an increase in computation time when the number of cells increases. However, when comparing the second largest (TM) and the largest (Zheng 68K) datasets, not all methods show an increase in computation time. Despite the increase in the number of cells between the two datasets, *CaSTLe*, *CHETAH*, and *SingleR* have a decreasing computation time. A possible explanation could be that the runtime of these methods also depends on the number of genes or the number of cell populations in the dataset. To evaluate the run time of the methods properly, we therefore investigated the effect of the number of cells, features, and cell populations separately (Fig. 7e–g).

To assess the effect of the number of genes on the computation time, we compared the computation time of the methods during the feature selection experiment (Fig. 7e). Most methods scale linearly with the number of genes. However, *LDA* does not scale very well when the number of genes increases. If the number of features is higher than the number of cells, the complexity of *LDA* is O(*g*^3), where *g* is the number of genes [44].

The effect of the number of cells on the timing showed that all methods increase in computation time when the number of cells increases (Fig. 7f). The differences in runtime on the largest dataset are larger. *scmapcluster*, for instance, takes 5 s to finish, while *Cell-BLAST* takes more than 11 h.

Finally, to evaluate the effect of the number of cell populations, the runtime of the methods on the AMB3, AMB16, and AMB92 datasets was compared (Fig. 7g). For most methods, this shows an increase in runtime when the number of cell populations increases, specially *singleCellNet*. For other methods, such as *ACTINN* and *scmapcell*, the runtime remains constant. Five classifiers, *scmapcell*, *scmapcluster*, *SVM*, *RF*, and *NMC*, have a computation time below 6 min on all the datasets.

## Discussion

**Fig. 8:**
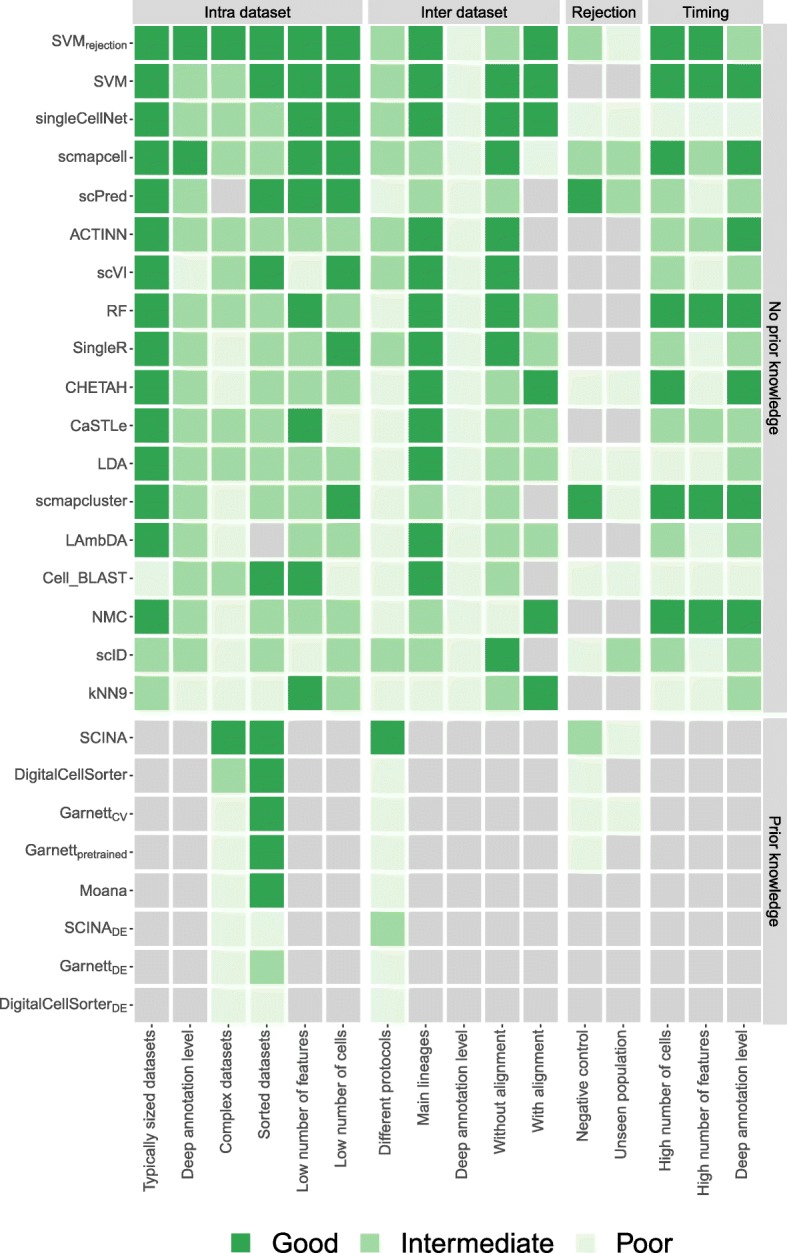
Summary of the performance of all classifiers during different experiments. For each experiment, the heatmap shows whether a classifier performs good, intermediate, or poor. Light gray indicates that a classifier could not be tested during an experiment. The gray boxes to the right of the heatmap indicate the four different categories of experiments: intra-dataset, inter-dataset, rejection, and timing. Experiments itself are indicated using the row labels. Additional file 1: Table S4 shows which datasets were used to score the classifiers exactly for each experiment. Gray boxes above the heatmap indicate the two classifier categories. Within these two categories, the classifiers are sorted based on their mean performance on the intra- and inter-dataset experiments

In this study, we evaluated the performance of 22 different methods for automatic cell identification using 27 scRNA-seq datasets. We performed several experiments to cover different levels of challenges in the classification task and to test specific aspects of the classifiers such as the feature selection, scalability, and rejection experiments. We summarize our findings across the different experiments (Fig. 8) and provide a detailed summary of which dataset was used for each experiment (Additional file 1: Table S4). This overview can be used as a user guide to choose the most appropriate classifier depending on the experimental setup at hand. Overall, several classifiers performed accurately across different datasets and experiments, particularly *SVM*_*rejection*_, *SVM*, *singleCellNet*, *scmapcell*, *scPred*, *ACTINN*, and *scVI*. We observed relatively lower performance for the inter-dataset setup, likely due to the technical and biological differences between the datasets, compared to the intra-dataset setup. *SVM*_*rejection*_, *SVM*, and *singleCellNet* performed well for both setups, while *scPred* and *scmapcell* performed better in the intra-dataset setup, and *scVI* and *ACTINN* had a better performance in the inter-dataset setup (Fig. 8). Of note, we evaluated all classifiers using the default settings. While adjusting these settings for a specific dataset might improve the performances, it increases the risk of overtraining.

Considering all three evaluation metrics (median F1-score, percentage of unlabeled cells, and computation time), *SVM*_*rejection*_ and *SVM* are overall the best-performing classifiers for the scRNA-seq datasets used. Although *SVM* has a shorter computation time, the high accuracy of the rejection option of *SVM*_*rejection*_, which allows flagging new cells and assigning them as unlabeled, results in an improved performance compared to *SVM*. Our results show that *SVM*_*rejection*_ and *SVM* scale well to large datasets as well as deep annotation levels. In addition, they did not suffer from the large number of features (genes) present in the data, producing the highest performance on the TM dataset using all genes, due to the incorporated L2 regularization. The comparable or higher overall performance of a general-purpose classier such as *SVM* warrants caution when designing scRNA-seq-specific classifiers that they do not introduce unnecessary complexity. For example, deep learning methods, such as *ACTINN* and *scVI*, showed overall lower performance compared to *SVM*, supporting recent observations by Köhler et al. [45].

*scPred* (which is based on an SVM with a radial kernel), *LDA*, *ACTINN*, and *singleCellNet* performed well on most datasets, yet the computation time is long for large datasets*. singleCellNet* also becomes slower with a large number of cell populations. Additionally, in some cases, *scPred* and *scmapcell/cluster* reject higher proportions of cells as unlabeled compared to *SVM*_*rejection*_, without a substantial improvement in the accuracy. In general, incorporating a rejection option with classification is a good practice to allow the detection of potentially novel cell populations (not present in the training data) and improve the performance for the classified cells with high confidence. However, for the datasets used in this study, the performance of classifiers with a rejection option, except for *SVM*_*rejection*_, did not show substantial improvement compared to other classifiers. Furthermore, our results indicate that designing a proper rejection option can be challenging for complex datasets (e.g., PBMC) and that relying on the posterior probabilities alone might not yield optimal results.

For datasets with deep levels of annotation (i.e., large number) of cell populations, the classification performance of all classifiers is relatively low, since the classification task is more challenging. *scVI*, in particular, failed to scale with deeply annotated datasets, although it works well for datasets with a relatively small number of cell populations. Further, applying the prior-knowledge classifiers becomes infeasible for deeply annotated datasets, as the task of defining the marker genes becomes even more challenging.

We evaluated the performance of the prior-knowledge methods (marker-based and pretrained) on PBMC datasets only, due to the limited availability of author-provided marker genes. For all PBMC datasets, the prior-knowledge methods did not improve the classification performance over supervised methods, which do not incorporate such prior knowledge. We extended some prior-knowledge methods such that the marker genes were defined in a data-driven manner using differential expression which did not improve the performance of these classifiers, except for *SCINA*_*DE*_ (with 20 marker genes) for the PbmcBench datasets. The data-driven selection of markers allows the prediction of more cell populations compared to the number of populations for which marker genes were originally provided. However, this data-driven selection violates the fundamental assumption in prior-knowledge methods that incorporating expert-defined markers improves classification performance. Further, several supervised classifiers which do not require markers to be defined a priori (e.g., *scPred* and *scID*) already apply a differential expression test to find the best set of genes to use while training the model. The fact that prior-knowledge methods do not outperform other supervised methods and given the challenges associated with explicit marker definition indicate that incorporating prior knowledge in the form of marker genes is not beneficial, at least for PBMC data.

In the inter-dataset experiments, we tested the ability of the classifiers to identify populations across different scRNA-seq protocols. Our results show that some protocols are more compatible with one another (e.g., 10Xv2 and 10Xv3), Smart-Seq2 is distinct from the other UMI-based methods, and CEL-Seq2 suffers from low replicability of cell populations across samples. These results can serve as a guide in order to choose the best set of protocols that can be used in studies where more than one protocol is used.

The intra-dataset evaluation included the Zheng sorted dataset, which consists of 10 FACS-sorted cell populations based on the expression of surface protein markers. Our results show relatively lower classification performance compared to other datasets, except the Zheng 68K dataset. The poor correlation between the expression levels of these protein markers and their coding genes mRNA levels [46] might explain this low performance.

Overall, we observed that the performance of almost all methods was relatively high on various datasets, while some datasets with overlapping populations (e.g., Zheng 68K dataset) remain challenging. The inter-dataset comparison requires extensive development in order to deal with technical differences between protocols, batches, and labs, as well as proper matching between different cell population annotations. Further, the pancreatic datasets are known to project very well across studies, and hence, using them to evaluate inter-dataset performance can be misleading. We recommend considering other challenging tissues and cell populations.

## Conclusions

We present a comprehensive evaluation of automatic cell identification methods for single-cell RNA sequencing data. Generally, all classifiers perform well across all datasets, including the general-purpose classifiers. In our experiments, incorporating prior knowledge in the form of marker genes does not improve the performance (on PBMC data). We observed large differences in the performance between methods in response to changing the input features. Furthermore, the tested methods vary considerably in their computation time which also varies differently across methods based on the number of cells and features.

Taken together, we recommend the use of the general-purpose *SVM*_*rejection*_ classifier (with a linear kernel) since it has a better performance compared to the other classifiers tested across all datasets. Other high-performing classifiers include *SVM* with a remarkably fast computation time at the expense of losing the rejection option, *singleCellNet*, *scmapcell*, and *scPred*. To support the future extension of this benchmarking work with new classifiers and datasets, we provide a Snakemake workflow to automate the performed benchmarking analyses (https://github.com/tabdelaal/scRNAseq_Benchmark/).

## Methods

### Classification methods

We evaluated 22 scRNA-seq classifiers, publicly available as R or Python packages or scripts (Table 1). This set includes 16 methods developed specifically for scRNA-seq data as well as 6 general-purpose classifiers from the scikit-learn library in Python [29]: linear discriminant analysis (*LDA*), nearest mean classifier (*NMC*), *k*-nearest neighbor (*kNN*), support vector machine (*SVM*) with linear kernel, SVM with rejection option (*SVM*_*rejection*_), and random forest (*RF*). The following functions from the scikit-learn library were used respectively: LinearDiscriminantAnalysis(), NearestCentroid(), KNeighborsClassifier(n_neighbors=9), LinearSVC(), LinearSVC() with CalibratedClassifierCV() wrapper, and RandomForestClassifier(n_estimators=50). For *kNN*, 9 neighbors were chosen. After filtering the datasets, only cell populations consisting of 10 cells or more remained. Using 9 neighbors would thus ensure that this classifier could also predict very small populations. For *SVM*_*rejection*_, a threshold of 0.7 was used on the posterior probabilities to assign cells as “unlabeled.” During the rejection experiments, also an LDA with rejection was implemented. In contrast to the LinearSVC(), the LinearDiscriminantAnalysis() function can output the posterior probabilities, which was also thresholded at 0.7.

scRNA-seq-specific methods were excluded from the evaluation if they did not return the predicted labels for each cell. For example, we excluded *MetaNeighbor* [47] because the tool only returns the area under the receiver operator characteristic curve (AUROC). For all methods, the latest (May 2019) package was installed or scripts were downloaded from their GitHub. For *scPred*, it should be noted that it is only compatible with an older version of Seurat (v2.0). For *CHETAH*, it is important that the R version 3.6 or newer is installed. For *LAmbDA*, instead of the predicted label, the posterior probabilities were returned for each cell population. Here, we assigned the cells to the cell population with the highest posterior probability.

During the benchmark, all methods were run using their default settings, and if not available, we used the settings provided in the accompanying examples or vignettes. As input, we provided each method with the raw count data (after cell and gene filtering as described in the “Data preprocessing” section) according to the method documentation. The majority of the methods have a built-in normalization step. For the general-purpose classifiers, we provided log-transformed counts, *log*_2_(*count* + 1).

Some methods required a marker gene file or pretrained classifier as an input (e.g., *Garnett*, *Moana*, *SCINA*, *DigitalCellSorter*). In this case, we use the marker gene files or pretrained classifiers provided by the authors. We did not attempt to include additional marker gene files for all datasets, and hence, the evaluation of those methods is restricted to datasets where a marker gene file for cell populations is available.

### Datasets

A total of 27 scRNA-seq datasets were used to evaluate and benchmark all classification methods, from which 11 datasets were used for intra-dataset evaluation using a cross-validation scheme, and 22 datasets were used for inter-dataset evaluation, with 6 datasets overlapping for both tasks as described in Table 2. Datasets vary across species (human and mouse), tissue (brain, pancreas, PBMC, and whole mouse), and the sequencing protocol used. The brain datasets, including Allen Mouse Brain (AMB), VISp, ALM (GSE115746), and MTG (phs001790), were downloaded from the Allen Institute Brain Atlas http://celltypes.brain-map.org/rnaseq. All 5 pancreatic datasets were obtained from https://hemberg-lab.github.io/scRNA.seq.datasets/ (Baron Mouse: GSE84133, Baron Human: GSE84133, Muraro: GSE85241, Segerstolpe: E-MTAB-5061, Xin: GSE81608). The CellBench 10X dataset was obtained from (GSM3618014), and the CellBench CEL-Seq2 dataset was obtained from 3 datasets (GSM3618022, GSM3618023, GSM3618024) and concatenated into 1 dataset. The Tabula Muris (TM) dataset was downloaded from https://tabula-muris.ds.czbiohub.org/ (GSE109774). For the Zheng sorted datasets, we downloaded the 10 PBMC-sorted populations (CD14+ monocytes, CD19+ B cells, CD34+ cells, CD4+ helper T cells, CD4+/CD25+ regulatory T cells, CD4+/CD45RA+/CD25− naive T cells, CD4+/CD45RO+ memory T cells, CD56+ natural killer cells, CD8+ cytotoxic T cells, CD8+/CD45RA+ naive cytotoxic T cells) from https://support.10xgenomics.com/single-cell-gene-expression/datasets; next, we downsampled each population to 2000 cells obtaining a dataset of 20,000 cells in total. For the Zheng 68K dataset, we downloaded the gene-cell count matrix for the “Fresh 68K PBMCs” [36] from https://support.10xgenomics.com/single-cell-gene-expression/datasets (SRP073767). All 13 PbmcBench datasets, 7 different sequencing protocols applied on 2 PBMC samples, were downloaded from the Broad Institute Single Cell portal https://portals.broadinstitute.org/single_cell/study/SCP424/single-cell-comparison-pbmc-data. The cell population annotation for all datasets was provided with the data, except the Zheng 68K dataset, for which we obtained the cell population annotation from https://github.com/10XGenomics/single-cell-3prime-paper/tree/master/pbmc68k_analysis. These annotations were used as a “ground truth” during the evaluation of the cell population predictions obtained from the classification methods.

### Data preprocessing

Based on the manual annotation provided in the datasets, we started by filtering out cells that were labeled as doublets, debris, or unlabeled cells. Next, we filtered genes with zero counts across all cells. For cells, we calculated the median number of detected genes per cell, and from that, we obtained the median absolute deviation (MAD) across all cells in the log scale. We filtered out cells when the total number of detected genes was below three MAD from the median number of detected genes per cell. The number of cells and genes in Table 2 represent the size of each dataset after this stage of preprocessing.

Moreover, before applying cross-validation to evaluate each classifier, we excluded cell populations with less than 10 cells across the entire dataset; Table 2 summarizes the number of cell populations before and after this filtration step for each dataset.

### Intra-dataset classification

For the supervised classifiers, we evaluated the performance by applying a 5-fold cross-validation across each dataset after filtering genes, cells, and small cell populations. The folds were divided in a stratified manner in order to keep equal proportions of each cell population in each fold. The training and testing folds were exactly the same for all classifiers.

The prior-knowledge classifiers, *Garnett*, *Moana*, *DigitalCellSorter*, and *SCINA*, were only evaluated on the Zheng 68K and Zheng sorted datasets, for which the marker gene files or the pretrained classifiers were available, after filtering genes and cells. Each classifier uses the dataset and the marker gene file as inputs and outputs the cell population label corresponding to each cell. No cross-validation is applied in this case, except for *Garnett* where we could either use the pretrained version (*Garnett*_*pretrained*_) provided from the original study, or train our own classifier using the marker gene file along with the training data (*Garnett*_*CV*_). In this case, we applied 5-fold cross-validation using the same train and test sets described earlier. Additional file 1: Table S1 shows the mapping of cell populations between the Zheng datasets and each of the prior-knowledge classifiers. For *Moana*, a pretrained classifier was used, this classifier also predicted cells to be memory CD8+ T cells and CD16+ monocytes, while these cell populations were not in the Zheng datasets.

### Evaluation of marker genes

The performance and choice of the marker genes per cell population per classifier were evaluated by comparing the F1-score of each cell population with four different characteristics of the marker genes across the cells for that particular cell population: (1) the number of marker genes, (2) the mean expression, (3) the average dropout rate, and (4) the average beta of the marker genes [37]. Beta is a score developed to measure how specific a marker gene for a certain cell population is based on binary expression.

### Selecting marker genes using differential expression

Using the cross-validation scheme, training data of each fold was used to select sets of 5, 10, 15, and 20 differentially expressed (DE) marker genes. First, if the data was not already normalized, a CPM read count normalization was applied to the data. Next, the data was log-transformed using *log*_2_(*count* + 1), and afterwards, the DE test could be applied. As recommended in [48], MAST was used to find the DE genes [49]. The implementation of MAST in the FindAllMarkers() function of Seurat v2.3.0 was used to do a one-vs-all differential expression analysis [50]. Genes returned by Seurat were sorted, and the top 5, 10, 15, or 20 significant genes with a positive fold change were selected as marker genes. These marker genes were then used for population prediction of the test data of the corresponding fold. These marker gene lists can be used by prior-knowledge classifiers such as *SCINA*, *Garnett*_*CV*_, and *DigitalCellSorter*, by modifying the cell type marker gene file required as an input to these classifiers. Such modification cannot be applied to the pretrained classifiers of *Garnett*_*pretrained*_ and *Moana*.

### Dataset complexity

To describe the complexity of a dataset, the average expression of all genes for each cell population (${\mathrm{avg}}_{C_i}$) in the dataset was calculated, representing the prototype of each cell population in the full gene space. Next, the pairwise Pearson correlation between these centroids was calculated $\underset{\forall i,j}{\mathrm{corr}}\left({\mathrm{avg}}_{C_i},{\mathrm{avg}}_{C_j}\right)$. For each cell population, the highest correlation to another cell population was recorded. Finally, the mean of these per cell population maximum correlations was taken to describe the complexity of a dataset.

$$ \mathrm{Complexity}=\mathrm{mean}\left(\underset{\forall i,i\ne j}{\max}\underset{\forall i,j}{\mathrm{corr}}\left({\mathrm{avg}}_{C_i},{\mathrm{avg}}_{C_j}\right)\right) $$

### Inter-dataset classification

#### CellBench

Both CellBench datasets, 10X and CEL-Seq2, were used once as training data and once as test data, to obtain predictions for the five lung cancer cell lines. The common set of detected genes by both datasets was used as features in this experiment.

#### PbmcBench

Using pbmc1 sample only, we tested all train-test pairwise combinations between all 7 protocols, resulting in 42 experiments. Using both pbmc1 and pbmc2 samples, for the same protocol, we used pbmc1 as training data and pbmc2 as test data, resulting in 6 additional experiments (10Xv3 was not applied for pbmc2). As we are now dealing with PBMC data, we evaluated all classifiers, including the prior-knowledge classifiers, as well as the modified versions of *SCINA*, *Garnett*_*CV*_, and *DigitalCellSorter*, in which the marker genes are obtained through differential expression from the training data as previously described. Through all these 48 experiments, genes that are not expressed in the training data were excluded from the feature space. Also, as these PbmcBench datasets differ in the number of cell populations (Table 2), only the cell populations provided by the training data were used for the test data prediction evaluation.

#### Brain

We used the three brain datasets, VISp, ALM, and MTG with two levels of annotations, 3 and 34 cell populations. We tested all possible train-test combinations, by either using one dataset to train and test on another (6 experiments) or using two concatenated datasets to train and test on the third (3 experiments). A total of 9 experiments were applied for each annotation level. We used the common set of detected genes between the datasets involved in each experiment as features.

#### Pancreas

We selected the four major endocrine pancreatic cell types (alpha, beta, delta, and gamma) across all four human pancreatic datasets: Baron Human, Muraro, Segerstolpe, and Xin. Additional file 1: Table S2 summarizes the number of cells in each cell type across all datasets. To account for batch effects and technical variations between different protocols, datasets were aligned using MNN [41] from the scran R package (version 1.1.2.0). Using both the raw data (unaligned) and the aligned data, we applied leave-one-dataset-out cross-validation where we train on three datasets and test on the left out dataset.

### Performance evaluation metrics

The performance of the methods on the datasets is evaluated using three different metrics: (1) For each cell population in the dataset, the F1-score is reported. The median of these F1-scores is used as a measure for the performance on the dataset. (2) Some of the methods do not label all the cells. These unassigned cells are not considered in the F1-score calculation. The percentage of unlabeled cells is also used to evaluate the performance. (3) The computation time of the methods is also measured.

### Feature selection

Genes are selected as features based on their dropout rate. The method used here is based on the method described in [22]. During feature selection, a sorted list of the genes is made. Based on this list, the top *n* number of genes can be easily selected during the experiments. First, the data is normalized using *log*_2_(*count* + 1). Next, for each gene, the percentage of dropouts, *d*, and the mean, *m*, of the normalized data are calculated. Genes that have a mean or dropout rate of 0 are not considered during the next steps. These genes will be at the bottom of the sorted list. For all other genes, a linear model is fitted to the mean and log2(*d*). Based on their residuals, the genes are sorted in descending order and added to the top of the list.

### Scalability

For the scalability experiment, we used the TM dataset. To ensure that the dataset could be downsampled without losing cell populations, only the 16 most abundant cell populations were considered during this experiment. We downsampled these cell populations in a stratified way to 1, 5, 10, 20, 50, and 100% of its original size (45,469 cells).

### Rejection

#### Negative control

Two human datasets, Zheng 68K and Baron Human, and two mouse datasets, AMB16 and Baron Mouse, were used. The Zheng 68K dataset was first stratified downsampled to 11% of its original size to reduce computation time. For each species, two different experiments were applied by using one dataset as a training set and the other as a test set and vice versa.

#### Unseen cell populations

Zheng 68K dataset was stratified downsampled to 11% of its original size to reduce computation time. Three different experiments were conducted. First, all cell populations that are a subpopulation of T cells were considered the test set. Next, the test set consisted of all subpopulations of CD4+ T cells. Last, only the CD4+/CD45RO+ memory T cells were in the test set. Each time, all cell populations that were not in the test set were part of the training set. Additional file 1: Table S3 gives an exact overview of the populations per training and test set.

### Benchmarking pipeline

In order to ensure reproducibility and support the future extension of this benchmarking work with new classification methods and benchmarking datasets, a Snakemake [51] workflow for automating the performed benchmarking analyses was developed with an MIT license (https://github.com/tabdelaal/scRNAseq_Benchmark/). Each tool (license permitting) is packaged in a Docker container (https://hub.docker.com/u/scrnaseqbenchmark) alongside the wrapper scripts and their dependencies. These images will be used through Snakemake’s singularity integration to allow the workflow to be run without the requirement to install specific methods and to ensure reproducibility. Documentation is also provided to execute and extend this benchmarking workflow to help researchers to further evaluate interested methods.

## Additional files

Additional file 1 Supplementary data, Tables S1-S4 and Figures S1–13. (PDF 12800 kb)

Additional file 2 Review history. (DOCX 42 kb)

## Data Availability

The filtered datasets analyzed during the current study can be downloaded from Zenodo (10.5281/zenodo.3357167). The source code is available in the GitHub repository, at https://github.com/tabdelaal/scRNAseq_Benchmark [52], and in the Zenodo repository, at 10.5281/zenodo.3369158 [53]. The source code is released under MIT license. Datasets accession numbers: AMB, VISp, and ALM [35] (GSE115746), MTG [31] (phs001790), Baron Mouse [30] (GSE84133), Baron Human [30] (GSE84133), Muraro [31] (GSE85241), Segerstolpe [32] (E-MTAB-5061), Xin [33] (GSE81608), CellBench 10X [34] (GSM3618014), CellBench CEL-Seq2 [34] (GSM3618022, GSM3618023, GSM3618024), TM [6] (GSE109774), and Zheng sorted and Zheng 68K [36] (SRP073767). The PbmcBench datasets [38] are not yet uploaded to any data repository.
