# Supplementary material for: A comparison of automatic cell identification methods for single-cell RNA sequencing data
Source: Genome Biol. 2019 Sep 9;20:194. doi: 10.1186/s13059-019-1795-z (PMC6734286; doi:10.1186/s13059-019-1795-z)
Supplement: Supplementary file 1 — Supplementary data, Tables S1-S4 and Figures S1–13. (PDF 12800 kb) [file 13059_2019_1795_MOESM1_ESM.pdf]

Supplementary Data for

A comparison of automatic cell identification methods for single-cell RNA-sequencing data

Tamim Abdelaal[1,2†] (t.r.m.abdelaal-1@tudelft.nl)

Lieke Michielsen[1,2†] (l.c.m.michielsen@student.tudelft.nl)

Davy Cats[3] (d.cats@lumc.nl)

Dylan Hoogduin[3] (ddhoogduin@gmail.com)

Hailiang Mei[3] (H.Mei@lumc.nl)

Marcel J.T. Reinders[1,2] (m.j.t.reinders@tudelft.nl)

Ahmed Mahfouz[1,2*] (a.mahfouz@lumc.nl)

[1] Leiden Computational Biology Center, Leiden University Medical Center, Einthovenweg 20, 2333ZC, Leiden, The Netherlands

[2] Delft Bioinformatics Lab, Delft University of Technology, Van Mourik Broekmanweg 6, 2628XE, Delft, The Netherlands

[3] Sequencing Analysis Support Core, Department of Biomedical Data Sciences, Einthovenweg 20, 2333ZC, Leiden University Medical Center, Leiden, The Netherlands

† Equal contribution

* Corresponding author (a.mahfouz@lumc.nl)

Table S1. Mapping of true cell population labels from PBMC datasets to cell population labels of the prior-knowledge classifiers.

<table>
<tr><th>True Labels</th><th>Garnett</th><th>DigitalCellSorter</th><th>Moana</th><th>SCINA</th></tr>
<tr><td>CD14+ Monocyte</td><td>CD14+ Monocyte</td><td>CD14+ Monocyte</td><td>CD14+ Monocyte</td><td>CD14+ Monocyte</td></tr>
<tr><td>Dendritic</td><td>Dendritic</td><td>Dendritic</td><td>Dendritic</td><td rowspan="2"></td></tr>
<tr><td>CD34+</td><td>CD34+</td><td></td><td></td></tr>
<tr><td>CD56+ NK</td><td>CD56+ NK</td><td>CD56+ NK</td><td>CD56+ NK</td><td>CD56+ NK</td></tr>
<tr><td>CD19+ B</td><td>CD19+ B</td><td>CD19+ B</td><td>CD19+ B</td><td>CD19+ B</td></tr>
<tr><td>CD4+ T Helper 2</td><td rowspan="4">CD4+ T cell</td><td rowspan="6">T cell</td><td rowspan="2"></td><td rowspan="8"></td></tr>
<tr><td>CD4+/CD25 T Reg</td></tr>
<tr><td>CD4+/CD45RA+/CD25- Naïve T</td><td>Naïve CD4+ T cells</td></tr>
<tr><td>CD4+/CD45RO+ Memory</td><td>Memory CD4+ T cells</td></tr>
<tr><td>CD8+ Cytotoxic T</td><td rowspan="2">CD8+ T cell</td><td></td></tr>
<tr><td>CD8+/CD45RA+ Naïve Cytotoxic</td><td>Naïve CD8+ T cells</td></tr>
<tr><td rowspan="2"></td><td rowspan="2"></td><td rowspan="2"></td><td>Memory CD8+ T cells</td></tr>
<tr><td>CD16+ Monocytes</td></tr>
</table>

Table S2. Cell type size for each pancreatic dataset used in the across dataset performance evaluation.

**Dataset**	**alpha**	**beta**	**delta**	**gamma**	**Total**
**Baron (Human)**	2326	2525	601	255	**5707**
**Muraro**	812	448	193	101	**1554**
**Segerstolpe**	872	263	110	195	**1440**
**Xin**	855	466	46	82	**1449**
**Total**	**4865**	**3702**	**950**	**633**	**10150**

Table S3. Cell populations in the training and test set during the rejection experiment applied on the Zheng 68K dataset.

	**Training set**	**Test set**
**T cells**	CD19+ B<br>CD56+ NK<br>Dendritic<br>CD14+ Monocyte<br>CD34+	CD8+ Cytotoxic T<br>CD8+/CD45RA+ Naive Cytotoxic T<br>CD4+/CD25 T reg<br>CD4+/CD45RO+ Memory T<br>CD4+/CD45RA+/CD25- Naive T<br>CD4+ T Helper
**CD4+ T cells**	CD19+ B<br>CD56+ NK<br>Dendritic<br>CD14+ Monocyte<br>CD34+<br>CD8+ Cytotoxic T<br>CD8+/CD45RA+ Naive Cytotoxic T	CD4+/CD25 T reg<br>CD4+/CD45RO+ Memory T<br>CD4+/CD45RA+/CD25- Naive T<br>CD4+ T Helper
**CD4+/CD45RO+ Memory T cells**	CD19+ B<br>CD56+ NK<br>Dendritic<br>CD14+ Monocyte<br>CD34+<br>CD8+ Cytotoxic T<br>CD8+/CD45RA+ Naive Cytotoxic T<br>CD4+/CD25 T reg<br>CD4+/CD45RA+/CD25- Naive T<br>CD4+ T Helper	CD4+/CD45RO+ Memory T

Table S4. Datasets used to score the performance of the classifiers per experiment in Figure 8.

Category	Experiment	Datasets
Intra dataset	Typically sized datasets	Baron Human<br>Baron Mouse<br>Xin<br>Muraro<br>Segerstolpe
	Deep annotation level	AMB92
	Complex datasets	Zheng 68K
	Sorted datasets	Zheng sorted
	Low number of features	TM with 100 features
	Low number of cells	TM downsampled to 1% (463 cells)
Inter dataset	Different protocols	Across PBMC benchmark
	Main lineages	Across brain - 3 levels of annotations
	Deep annotation level	Across brain - 34 levels of annotations
	Without alignment	Across pancreas - unaligned
	With alignment	Across pancreas - aligned
Rejection	Negative control	Downsampled Zheng 68K<br>Baron Human<br>AMB16<br>Baron Mouse
	Unseen population	Downsampled Zheng 68K
Timing	High number of cells	TM with most abundant 16 populations (45,469 cells)
	High number of features	TM with all features
	Deep annotation level	AMB92

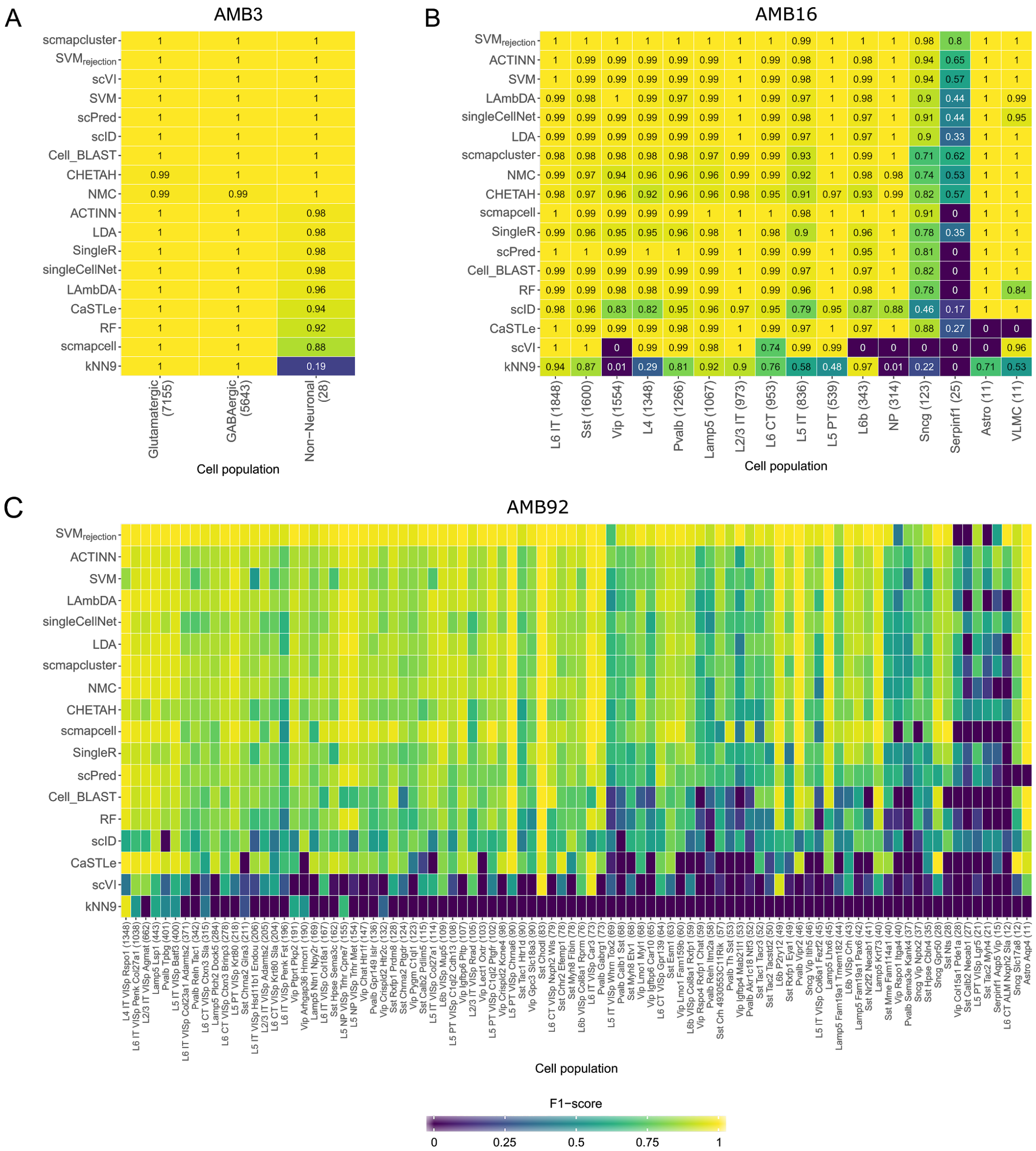

Figure S1. Classification performance across different annotation levels in the Allen Mouse Brain dataset. Heatmaps show the F1-scores of each classifier for each cell population in the **(A)** AMB3, **(B)** AMB16, and **(C)** AMB92 datasets. The cell populations are sorted from left-to-right in descending order according to their size (i.e. number of cells). The size of each population is indicated between brackets. In each heatmap, the classifiers are sorted according to their mean performance across all cell populations.

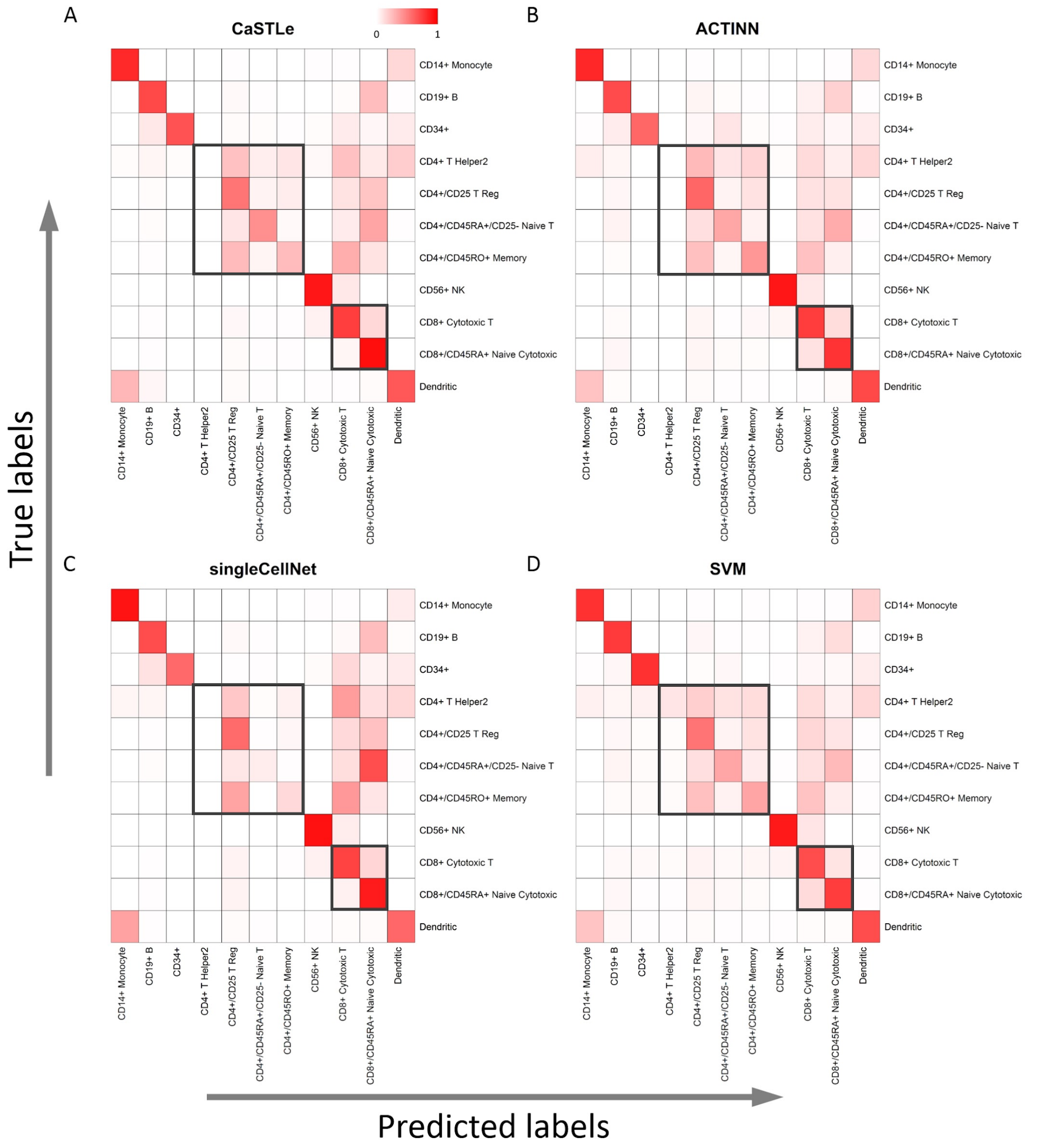

Figure S2. Confusion matrices for the Zheng 68K dataset. Results of four classifiers, **(A)** *CaSTLe*, **(B)** *ACTINN*, **(C)** *singleCellNet*, and **(D)** *SVM*, are shown. Rows indicate the true labels and columns indicate the predicted labels. Each cell in the heatmap is colored according to the percentage of overlapping cells between the true and predicted cell population. Black boxes highlight the four subpopulations of CD4 and the two subpopulations of CD8 T-cells.

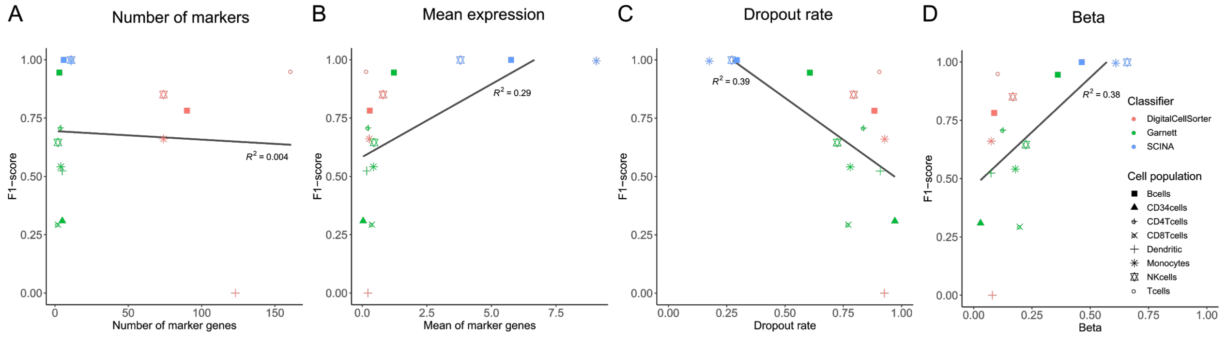

Figure S3. Effect of marker-genes on the performance of the classifiers. Scatterplots compare the **(A)** number of marker-genes, **(B)** mean expression, **(C)** dropout rate, and **(D)** beta, a measure for the specificity, with the performance of the marker based classifiers. Different classifiers are indicated with different colors, different cell populations with different shapes.

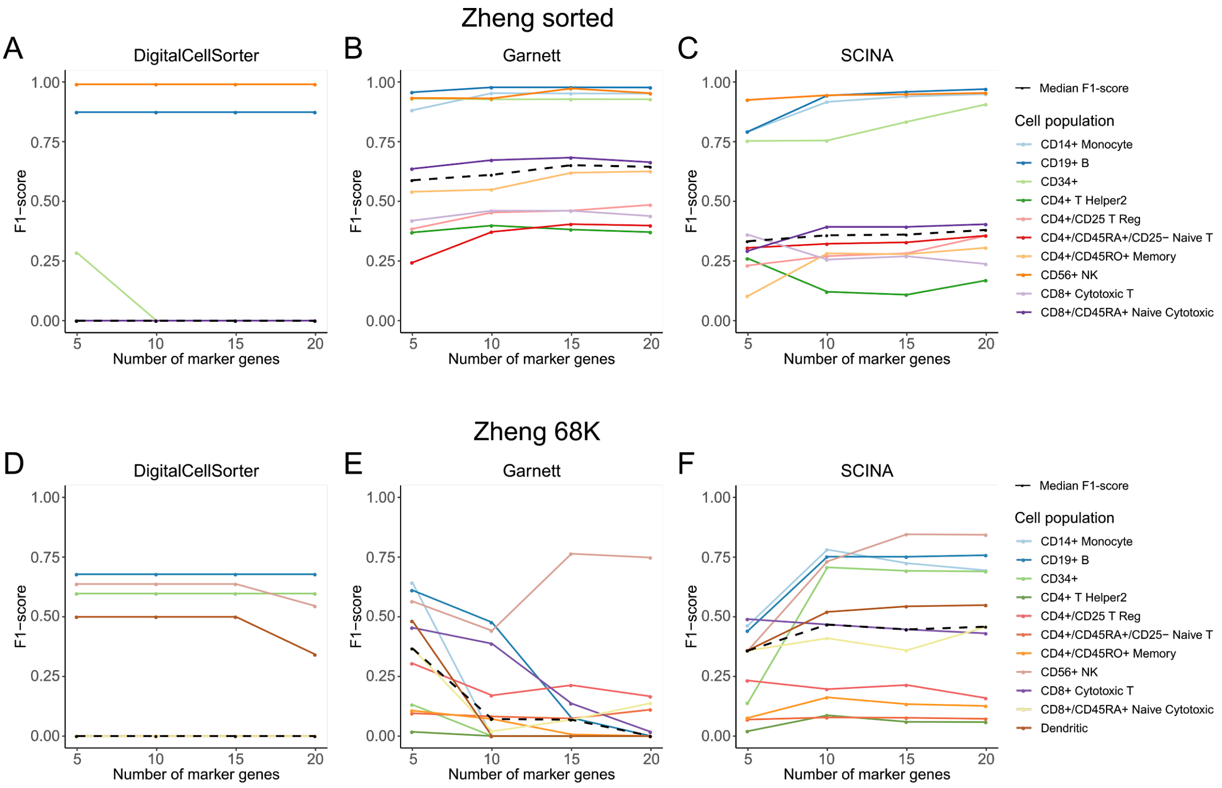

Figure S4. Performance of marker-based classifiers using differentially expressed genes. Line plots show the performance of marker-based classifiers using different number of marker-genes on the **(A-C)** Zheng sorted and **(D-F)** Zheng 68K dataset. marker-genes were selected using differential expression. Different cell populations are indicated using different colors. The median F1-score of the classifier is indicated using a dashed black line.

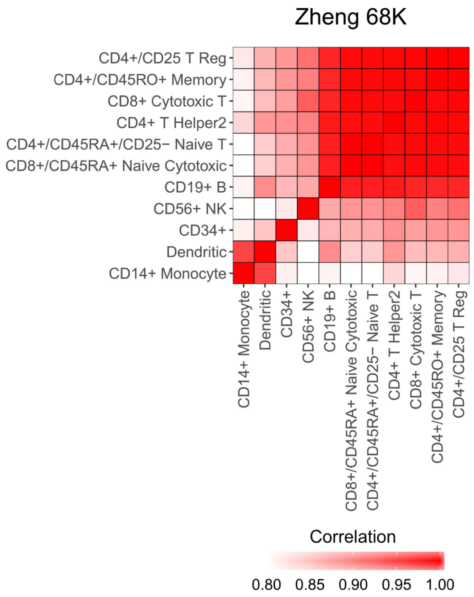

Figure S5. Correlation between cell populations in the Zheng 68K dataset. Heatmap showing the pairwise Pearson correlation between the different cell populations in the Zheng 68K dataset.

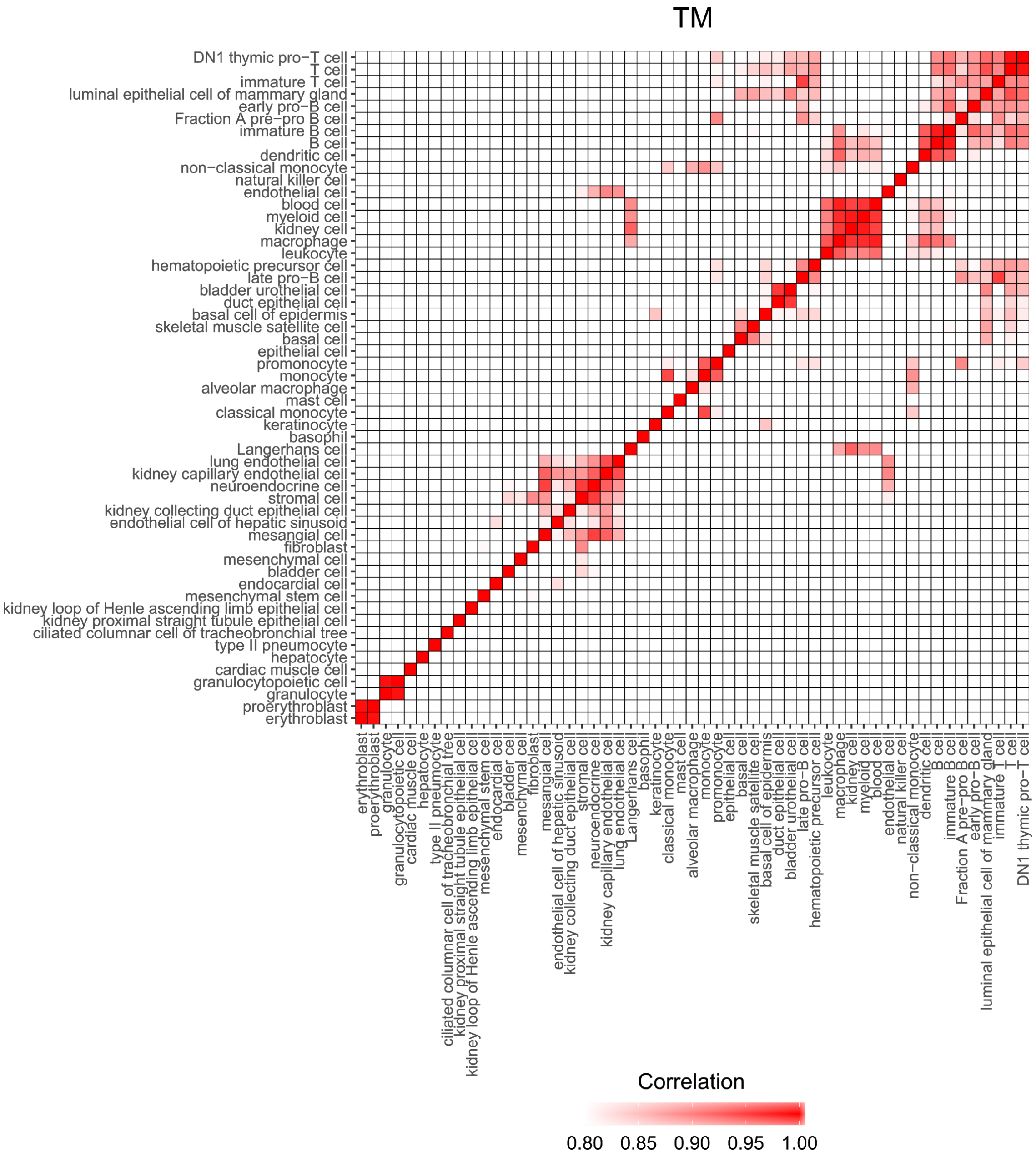

Figure S6. Correlation between cell populations in the TM dataset. Heatmap showing the pairwise Pearson correlation between the different cell populations in the TM dataset.

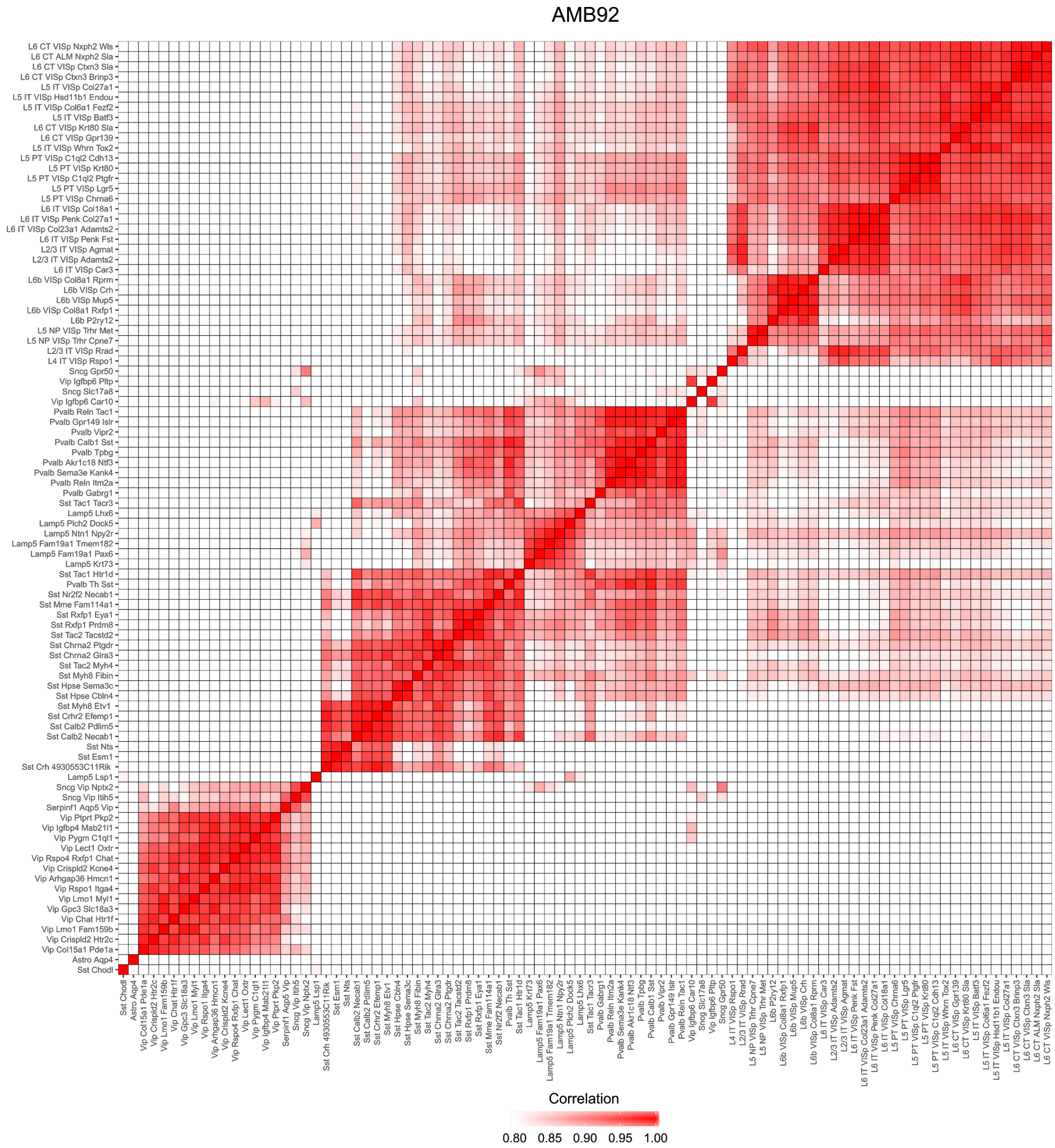

Figure S7. Correlation between cell populations in the AMB92 dataset. Heatmap showing the pairwise Pearson correlation between the different cell populations in the AMB92 dataset.

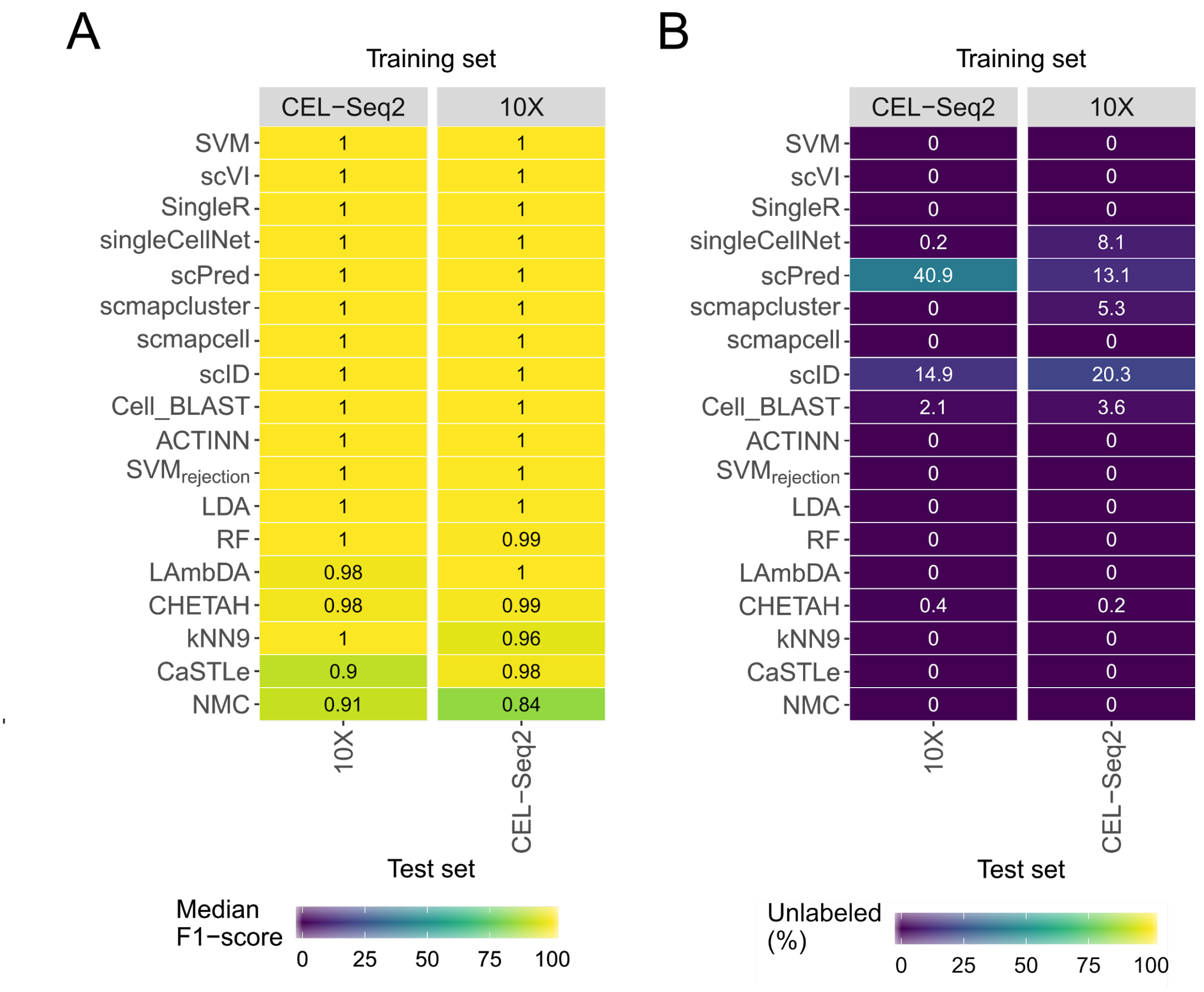

Figure S8. Classification performance across the CellBench datasets. Heatmaps show the **(A)** median F1-score and **(B)** percentage of unlabeled cells across the CellBench datasets. The training set is indicated above the heatmap, the test set below. Classifiers are sorted based on their mean performance in **(A).**

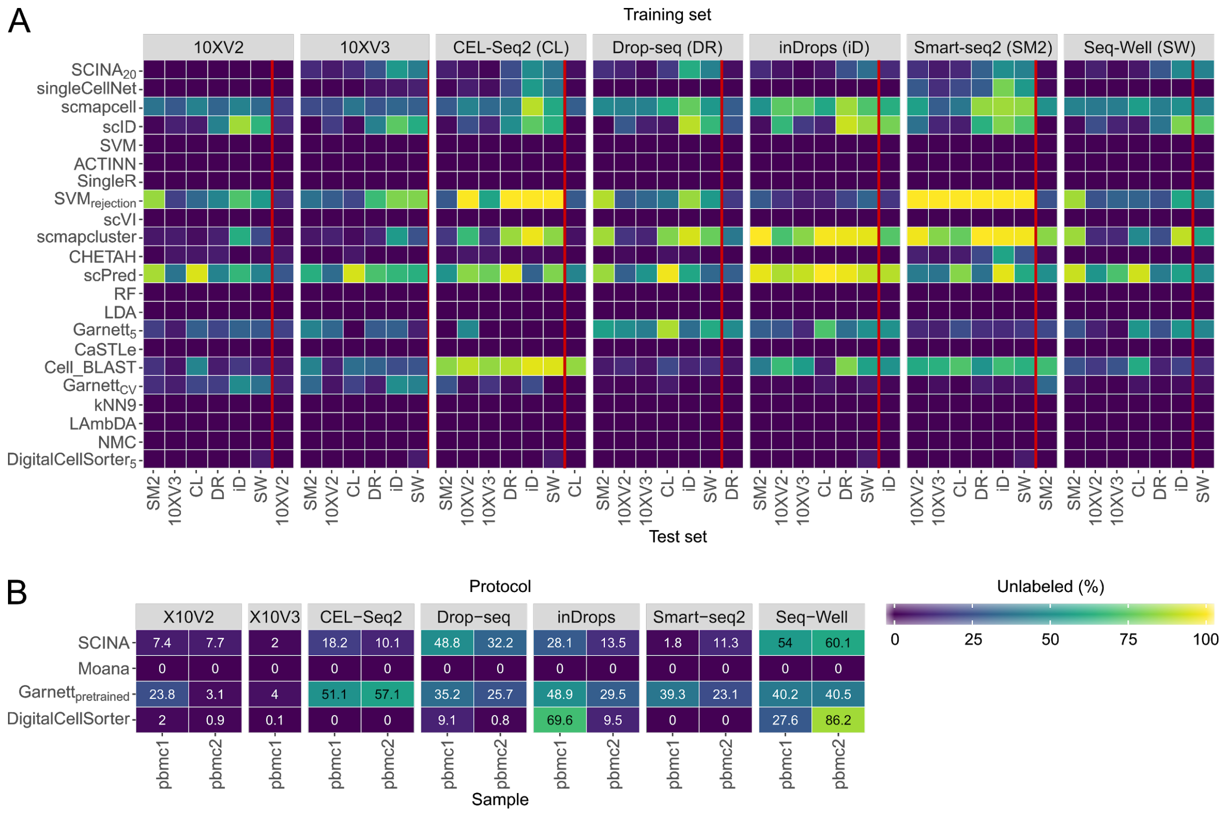

Figure S9. Percentage of unlabeled cells across the PbmcBench datasets. (A) Heatmap showing the median F1-score of the supervised classifiers for all train-test pairwise combination across different protocols. The training set is indicated in the grey box on top of the heatmap, the test set is indicated using the column labels below. Results showed to the left of the red line represent the comparison between different protocol using sample pbmc1. Results showed to the right of the red line represent the comparison between different samples using the same protocol, with pbmc1 used for training and pbmc2 used for testing. For *SCINA,*, *Garnett*$_{DE}$, and *DigitalCellSorter*$_{DE}$ different numbers of marker-genes were tested. Only the best result is shown here. **(B)** Percentage of unlabeled of the prior-knowledge classifiers on both samples of the different protocols. The protocol is indicated in the grey box on top of the heatmap, the sample is indicated with the labels below. Classifiers in the heatmaps are ordered based on their mean performance in Figure 3.

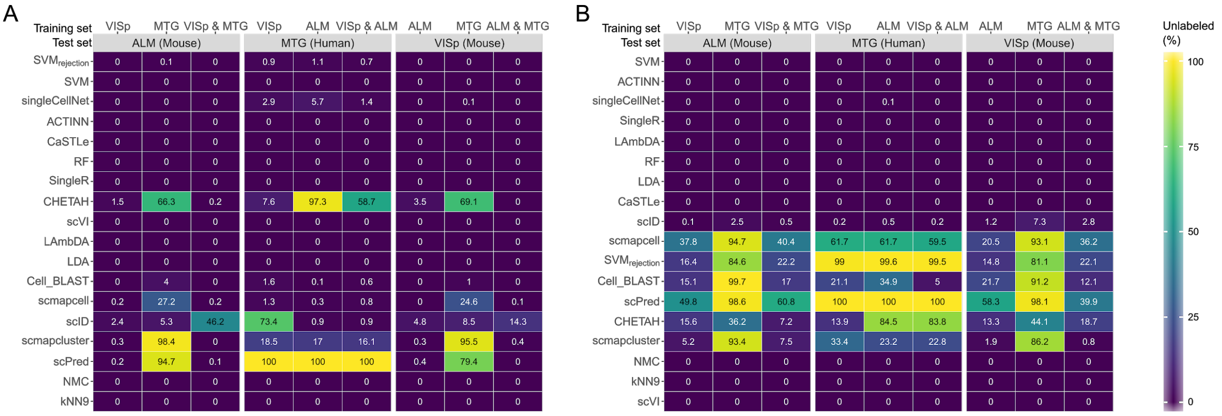

Figure S10. Percentage of unlabeled across brain datasets. Heatmaps show the percentage of unlabeled of the classifiers on **(A)** major lineage annotation with three cell populations, and **(B)** deeper level of annotation with 34 cell populations. The training set(s) are indicated using the column labels on top of the heatmap. The test set is indicated in the grey box. In each heatmap the classifiers are ordered based on their mean performance in Figure 4.

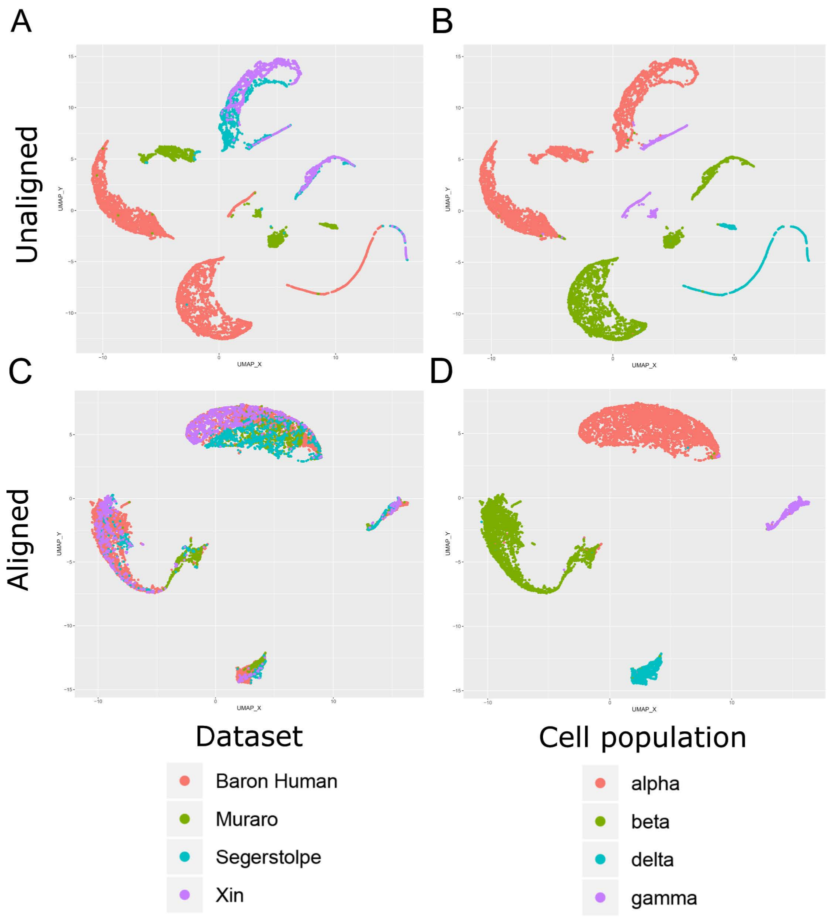

Figure S11. UMAP plots of the four pancreatic datasets used in the inter-dataset experiment. (A-B) UMAP plots before and **(C-D)** after alignment using MNN. In **(A, C)** the cells are colored by dataset and in **(B, D)** the cells are colored by cell population.

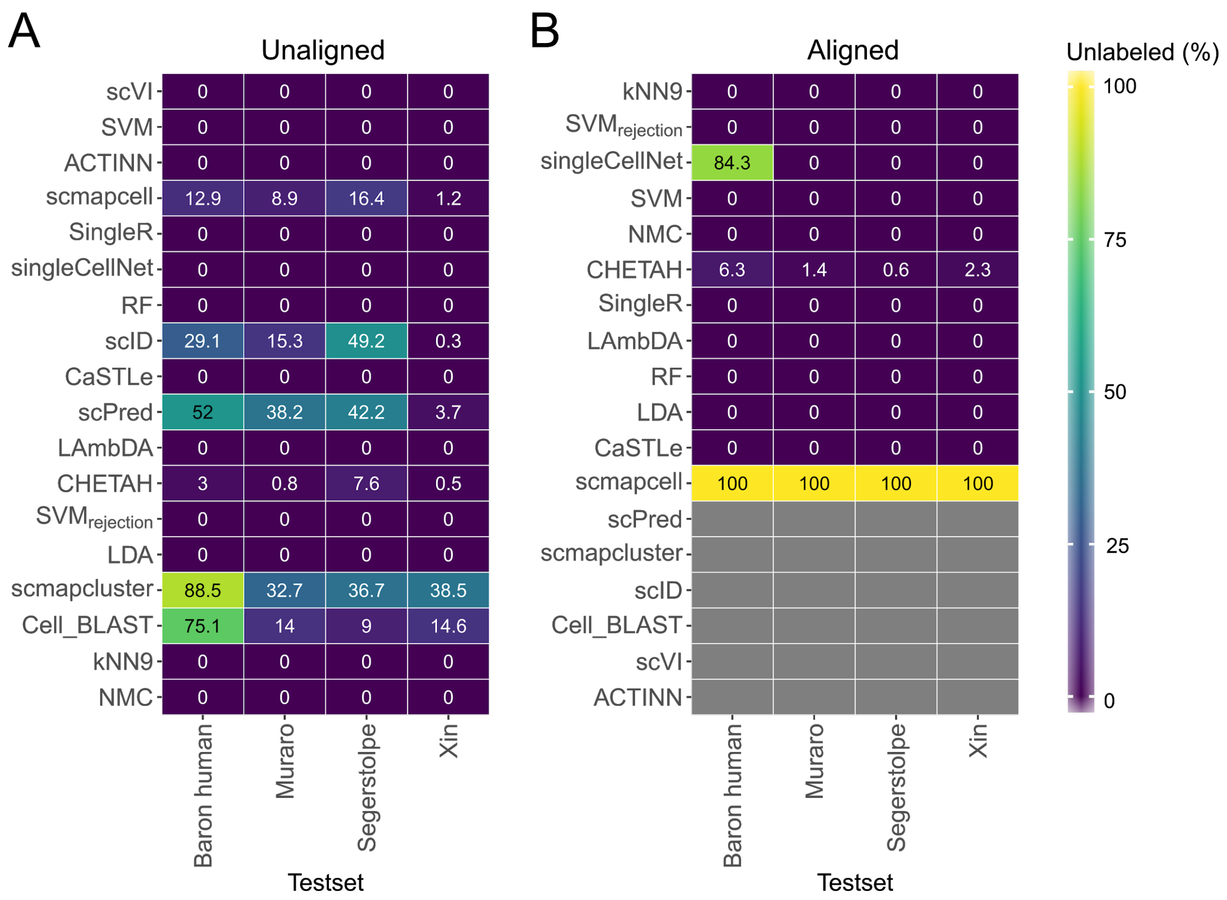

Figure S12. Percentage of unlabeled cells across different pancreatic datasets. Heatmaps showing the percentage of unlabeled for each classifier for the **(A)** unaligned and **(B)** aligned datasets. The column labels indicate which of the four datasets was used as a test set, in which case the other three sets were used as training data. Grey boxes indicate that the corresponding method could not be tested on the corresponding dataset. In each heatmap, the classifiers are ordered based on their mean performance in Figure 5.

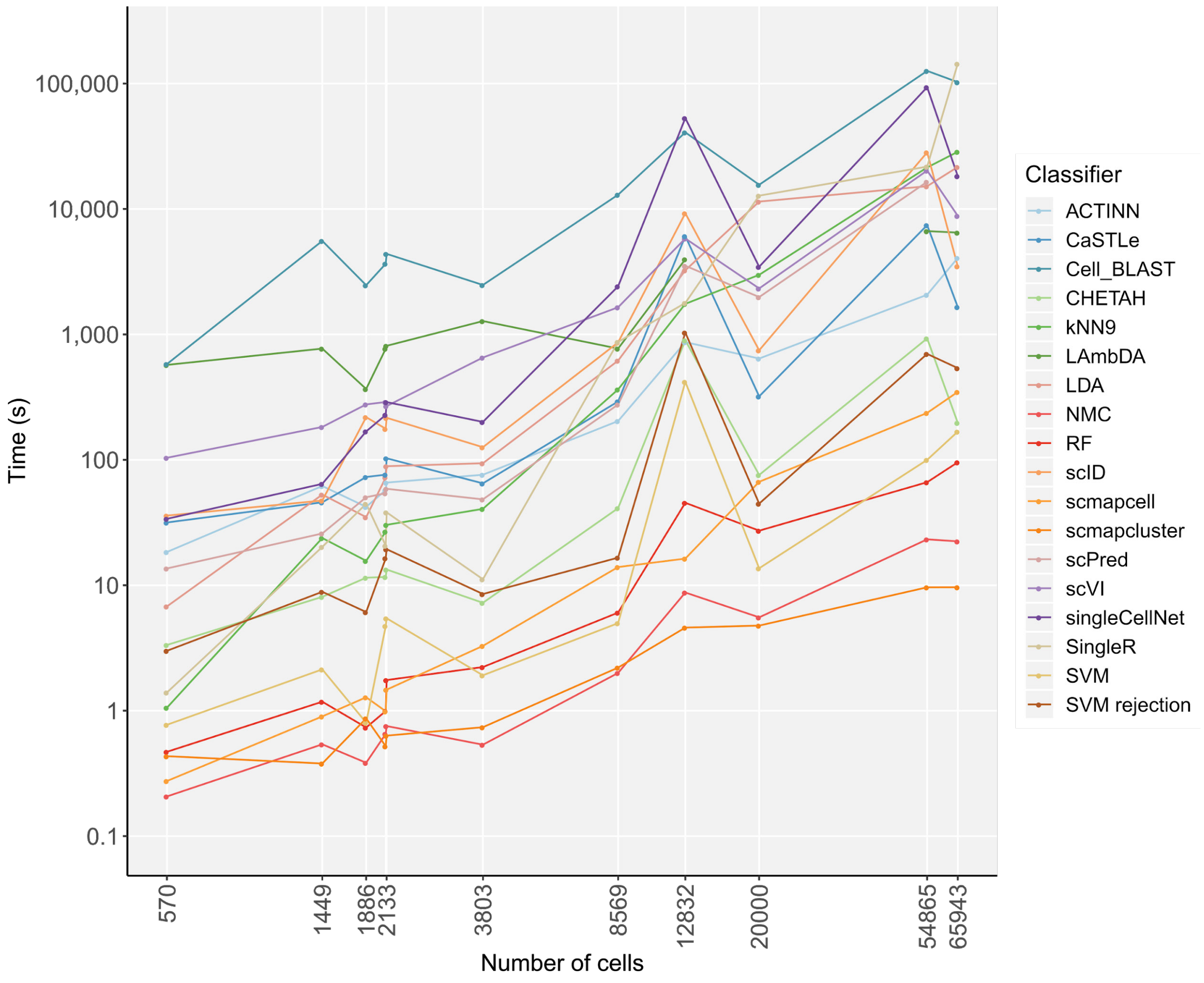

Figure S13. Computation time across different datasets. Line plots showing the computation time of the classifiers with the number of cells in all datasets. Classifiers are indicated using different colors. Both axes in the plot are log-scaled.
