# Supplementary material for: A comparison of automatic cell identification methods for single-cell RNA sequencing data
Source: Genome Biol. 2019 Sep 9;20:194. doi: 10.1186/s13059-019-1795-z (PMC6734286; doi:10.1186/s13059-019-1795-z)
Supplement: Supplementary file 2 — Review history. (DOCX 42 kb) [file 13059_2019_1795_MOESM2_ESM.docx]

Review History

**Comments to author and authors’ point-by-point responses to the reviewers’ comments:**

**Reviewer 1**

The authors address the important and timely problem of assigning cell type labels to cells investigated with scRNA-seq. They benchmark 20 different algorithms for this purpose, some designed for this task and more general-purpose classifiers on 8 different scRNA-seq datasets. The authors measure performance against the existing annotation for these datasets.

Next to performance, running time and sensitivity to input features are also investigated. In general, they find that most classifiers perform well and that the general-purpose SVM classifier excels with overall best performance across different datasets. I think benchmarking the performance of automatic cell type identification is vital for the future of single cell analysis, as it currently presents the biggest bottleneck during analysis. However, I disagree with some of their conclusions and have further suggestions to improve the work presented.

1. The authors present two evaluations of the methods one where the same dataset is split and also used as training data and one evaluation across datasets. The first evaluation is highly artificial and obviously biased. For the conclusions of this first analysis to be useful, the reader needs to know how different they are from the conclusions arrived to in the second evaluation.

*Response: We agree with the reviewer that the inter-dataset (across datasets) evaluation is a more realistic and practical scenario, in which a reference dataset (e.g. atlas) can be used to annotate cells in a new (unannotated) dataset. However, in order to perform well across datasets, a classifier should first perform well when tested using the intra-dataset setup (within dataset using cross validation) on the reference dataset. In addition, using this intra-dataset setup allows us to fairly evaluate different aspects of the classification process (e.g. feature selection, scalability and annotation levels) regardless of the technical and biological differences across datasets. These aspects are better evaluated in the ideal and artificial situation using the intra-dataset setup. We do, however, agree that the inter-dataset setup is more informative about practical use. We have now expanded out inter-dataset evaluation by including a number of additional datasets (13 PbmcBench datasets, three brain datasets and two CellBench datasets).*

*We acknowledge the importance of clearly distinguishing between the conclusions drawn from each experimental setup. Comparing both setups, our results show that some classifiers perform well in both setups (SVM rejection, SVM and singleCellNet), some perform better for the intradataset setup (scPred and scmapcell), while other classifiers perform better for the inter-dataset setup (scVI and ACTINN). We now added these conclusions to the Discussion section. Further, we added a new Figure 8 to summarize the performance of all classifiers across all experiments carried out in the study. In the figure, we distinguish between the performance on intra- and interdataset evaluation tasks. Finally, we added Supplementary Table 4 to clearly indicate which dataset(s) were used in each experiment stated in Figure 8.*

*Changes to the manuscript:*

*• We now clearly distinguish between the intra-dataset and inter-dataset setup in the Results and the Methods section.*

*• We modified the following paragraph in the Background section:*

*“Here, we benchmarked 22 classification methods to automatically assign cell identities including single cell-specific and general-purpose classifiers. The methods were evaluated using 27 publicly available single cell RNA-sequencing datasets of different sizes, technologies, species, and complexity. The performance of the methods was evaluated based on their accuracy, percentage of unclassified cells, and computation time. We performed several experiments to cover different levels of challenge in the classification task, and to test specific features or tasks such as the feature selection, scalability and rejection experiments. We evaluated the classification performance through two experimental setups, 1) intra-dataset in which we applied 5-fold crossvalidation within each dataset, and 2) inter-dataset involving across datasets comparisons. The inter-dataset comparison is more realistic and more practical, where a reference dataset (e.g. atlas) is used to train a classifier which can then be applied to identify cells in new unannotated datasets. However, in order to perform well across datasets, the classifier should also perform well using the intra-dataset setup on the reference dataset. The intra-dataset experiments , albeit artificial, provide an ideal scenario to evaluate different aspects of the classification process (e.g. feature selection, scalability and different annotation levels), regardless of the technical and biological variations across datasets. In general, most classifiers perform well across all datasets in both experimental setups (inter- and intra-dataset), including the general-purpose classifiers. In our experiments, incorporating prior knowledge in the form of markergenes does not improve the performance. We observed large variation across different methods in the computation time and classification performance in response to changing the input features and the number of cells. Our results highlight the general-purpose support vector machine (SVM) classifier as the best performer overall.” • We modified the following paragraph in the Discussion section: “In this study, we evaluated the performance of 22 different methods for automatic cell identification using 27 scRNA-seq datasets. We performed several experiments to cover different levels of challenges in the classification task, and to test specific aspects of the classifiers such as the feature selection, scalability and rejection experiments. We summarize our findings across the different experiments (Figure 8) and provide a detailed summary of which dataset was used for each experiment (Supplementary Table 4). This overview can be used as a user-guide to choose the most appropriate classifier depending on the experimental setup at hand. Overall, several classifiers performed accurately across different datasets and experiments, particularly: SVMrejection, SVM, singleCellNet, scmapcell, scPred, ACTINN and scVI. We observed relatively lower performance for the inter-dataset setup, likely due to the technical and biological differences between datasets, compared to the intra-dataset setup. SVMrejection, SVM and singleCellNet performed well for both setups, while scPred and scmapcell performed better in the intra-dataset setup, and scVI and ACTINN had better performance in the inter-dataset setup (Figure 8).”*

2. The authors' conclusion that marker-based algorithms perform worse than reference-based algorithms cannot be made. Unlike the reference-based algorithms, the marker-based algorithms were not trained on the dataset itself. Thus they had no chance to learn about dataset specific noise and hence are disadvantaged. A better comparison would be with marker-based algorithms that are also trained on the same data (as suggested in the next point).

*Response: We agree with the reviewer that the supervised methods trained on the dataset itself do get the chance to learn dataset specific noise and features, and thus might have an advantage over the marker-based classifiers. It is important to note that the marker-based classifiers have no training procedure, as these methods either (1) use a pre-trained model (e.g. Garnettpretrained and Moana) which we directly use to obtain cell identities for the test data, or (2) predict cell identities by fitting the expression of the test data to the defined cell population markers (e.g. DigitalCellSorter and SCINA). Garnett is the only exception since we could also retrain our own classifier as we did (GarnettCV). In this case, GarnettCV tries to find representative cells, from the training data, for the cell populations described in the marker-genes file. Thus, Garnett does not completely learn from the training data itself in the sense that the markers were defined apriori. As such, the developers of the marker-based classifiers choose to benefit from expert-defined markers, rather than learn the cell identities from the training data. In the publications and preprints of all four marker-based classifiers that we evaluated, there are no recommendations on how to pick cell type markers.*

*That being said, this disadvantage does not hold anymore in inter-dataset experiments. We now added an inter-dataset evaluation for all classifiers, including the marker-based methods, using the PbmcBench datasets. In this experiment, the supervised classifiers learn the cell populations from one dataset and produce prediction to a different dataset, and thus they do not have this dataset specific advantage over the marker-based classifiers anymore. We observed that, except for SCINA using differential expressed genes (see point 3 and 4), as for the intra-dataset experiment, the marker-based classifiers do not outperform other supervised classifiers (Figure 3).*

*Further, given that the choice of the marker-genes is a crucial part of marker-based classification, we evaluated the marker gene lists provided by Garnett, SCINA and DigitalCellSorter. The marker-genes provided by these three classifiers differ greatly for the same cell population (e.g. only one marker was common across all classifiers across all cell populations). We evaluated the effect of four parameters on the classification performance in terms of median F1-score: 1) number of genes, 2) mean expression, 3) dropout rate, and 4) a measure of specificity of a marker gene for the corresponding population, called beta (Hodge et al. 2018). We observed that the marker-genes with low dropout rate and high beta yield higher performance (Supplementary Figure 3).*

*Regarding the reviewer’s remark on training the marker-based methods on the data, we refer to the answer in reaction to comments number 3 and 4 from the reviewer.*

*Changes to the manuscript:*

*• We added the new PbmcBench datasets to fairly evaluate all classifiers, including the marker-based, across datasets.*

*• We added the following paragraph to the Results section - Performance evaluation across datasets:*

*“We also tested the prior-knowledge classifiers on all 13 PbmcBench datasets. The prior-knowledge classifiers showed lower performance compared to other classifiers (average median F1-score < 0.6), with the exception of SCINA which was only tested on three cell populations (Figure 3B, Supplementary Figure 9B). These results are in line with our previous conclusions from the Zheng sorted and Zheng 68K datasets in the intra-dataset evaluation.”*

*• We added the following paragraph to the Methods section - Inter-dataset classification: “PbmcBench. Using pbmc1 sample only, we tested all train-test pairwise combinations between all seven protocols, resulting in 42 experiments. Using both pbmc1 and pbmc2 samples, for the same protocol we used pbmc1 as training data and pbmc2 as test data, resulting in six additional experiments (10Xv3 was not applied for pbmc2). As we are now dealing with PBMC data, we evaluated all classifiers, including the prior-knowledge classifiers, as well as the modified versions of SCINA, GarnettCV and DigitalCellSorter, in which the marker-genes are obtained through differential expression from the training data as previously described. Through all these 48 experiments, genes that are not expressed in the training data were excluded from the feature space. Also, as these PbmcBench datasets differ in the number of cell populations (Table 2), only cell populations provided by the training data were used for the test data prediction evaluation.”*

*We added the following paragraph to the Results section - The performance of priorknowledge classifiers strongly depends on the selected marker-genes:*

*“Some prior-knowledge classifiers, SCINA, DigitalCellSorter and GarnettCV, used markergenes to classify the cells. For the PBMC datasets, the number of marker-genes per cell population varies across classifiers (2–161 markers) and the marker-genes show very little overlap. Only one B cell marker gene, CD79A, is shared by all classifiers while none of the marker-genes for the other cell populations is shared by the three classifiers. We analyzed the effect of the number of marker-genes, mean expression, dropout rate, and the specificity of each marker gene (beta score, see Methods), on the performance of the classifier (Supplementary Figure 3). The dropout rate and marker specificity (betascore) are strongly correlated with the median F1-score, highlighting that the performance does not only depend on biological knowledge, but also on technical factors.”*

*• We added the following paragraph to the Methods section - Evaluation of marker-genes:*

*“The performance and choice of the marker-genes per cell population per classifier were evaluated by comparing the F1-score of each cell population with four different*

*characteristics of the marker-genes across the cells for that particular cell population: 1) the number of marker-genes, 2) the mean expression, 3) the average dropout rate, and 4) the average beta of the marker-genes [26]. Beta is a score developed to measure how specific a marker gene for a certain cell population is based on binary expression.”*

*• Added Figure 3 and Supplementary Figure 9.*

*• Added Supplementary Figure 3.*

3. I disagree with the authors that the evaluation of the marker-based algorithms was only possible on the Zheng et al dataset. I think approaching this problem with the same 5-fold crossvalidation approach, used for the evaluation of the other methods, can work. One would use 4- folds for finding marker genes given the annotation and 1-fold for performance evaluation.

4. It would be good to evaluate the marker-based methods' sensitivity to the number of marker genes provided.

*Response to point 3 and 4 (related points):*

*Following up on point 2, we believe that the selection of the marker-genes is a fundamental part for the marker-based classifiers. As such, learning the marker-genes as suggested by the reviewer is counterintuitive to the main idea behind the marker-based methods, which depend on the expert knowledge or literature to define these marker-genes. We reiterate that in the original publications/preprints of the marker-based methods, the cell population markers are expert defined and there are no recommendations as to how these could be defined in a data-specific manner. And as mentioned earlier, there is no training step for marker-based classifiers (see response to point #2). Finding markers through training (based on differential expression for*

*example) would, in our opinion, seem like designing a new classifier. In fact, some of the supervised classifiers we tested (such as scPred and scID) indeed apply differential expression during the training process to select the best marker-genes to train their model.*

*Nevertheless, to evaluate whether learning the marker-genes from the data improves the performance of the marker-based classifiers and to obtain a better comparison with the other supervised classifiers, we tested the suggested scheme to find markers from the data using differential expression. We provide these markers to the marker-based classifiers and evaluate their performance. We applied this process only to the PBMC datasets so we can compare the performance with the markers provided already from the authors. This includes the Zheng 68K and Zheng sorted (new dataset added, more elaboration in point 5) datasets for intra-dataset experiment, and the PbmcBench datasets for inter-dataset experiment.*

*To find the marker-genes, we applied a differential expression test on the training data using MAST. If no normalization is already applied, training data was first normalized to CPM (count per million) and log transformed. We used the Seurat implementation to apply MAST using the one-vs-all setup. For the intra-dataset experiment, the training data consists of the 4-folds used for training, which means the marker-genes can differ slightly between folds. For the interdataset experiment, the training data is the one dataset used as training, thus the differential expression test is applied only once.*

*We applied this experiment for Garnett, SCINA and DigitalCellSorter (Moana is a hard-coded pre-trained classifier). Selecting the markers from the data did not improve the classification performance over the supervised classifiers, except for SCINA on the PbmcBench datasets when using 20 marker-genes per population (Figure 1 and 3). However, this data-driven selection allowed these marker-based classifiers to predict more populations, as the original marker-genes list provided by the authors did not cover all PBMC populations (summarized in Supplementary Table 1). So in part, this drop in performance can be attributed to testing the classifiers on deeper (and more challenging) annotations. Further, this data-driven selection of the marker-genes produced lower performance compared with the original markers, although this comparison is not straightforward, since the original markers consider less cell populations. However, If we consider the three cell populations that the original SCINA can predict (B cells, Monocytes and NK cells), the data-driven version of SCINA (which uses the differentially expressed marker-genes) still does not improve over the original SCINA (median F1-score of 0.95 vs 1.0, respectively, on the Zheng sorted dataset). This result shows that these marker based classifiers are best fitted to the expert markers defined by the authors. As a result, we decided not to test these marker-based classifiers further on other (non-PBMC) datasets for which the authors do not provide mark genes.*

*In response to point 4, we tested the classification performance when changing the number of marker-genes. We used the top 5, 10, 15, 20 differentially expressed genes per population. The results did not show a clear pattern as the performance changes per dataset and per classifier as shown in Supplementary Figure 4.*

*Changes to the manuscript:*

*• We added the following paragraph to the Results section - The performance of prior knowledge classifiers strongly depends on the selected marker-genes:*

*“The difference between the marker-genes used by each method underscores the challenge of marker-genes selection, especially for smaller cell populations. Moreover, public databases of cell type markers (e.g. PanglaoDB [16] and CellMarker [17]) often provide different markers for the same population. For example, CellMarker provides 33 marker-genes for B cells, while PanglaoDB provides 110 markers, with only 11 marker-genes overlap between the two databases.Given the differences between “expert-defined” markers and the correlation of classification performance and technical dataset-specific features (e.g. dropout rate), we tested if the performance of prior knowledge methods can be improved by automatically selecting marker-genes based on differential expression. Through the cross-validation scheme, we used the training folds to select the marker-genes of each cell population based on differential expression (see Methods) and later used these markers to evaluate the classifiers’ performance on the testing fold. We tested this approach on the two PBMC datasets, Zheng sorted and Zheng 68K for different numbers of marker-genes (5, 10, 15, and 20 markers). In Figure 1, the best result across the number of markers for SCINADE, GarnettDE, and DigitalCellSorterDE are shown.The median F1-score obtained using the differential expression-defined markers is significantly lower compared to the original versions of classifiers using the markers defined by the authors. This lower performance is in part due to the low performance on challenging populations, such as subpopulations of CD4+ and CD8+ T cell populations (F1-score ≤ 0.68) (Supplementary Figure 4). These challenging populations are not identified by the original classifiers since the markers provided by the authors only considered annotations at a higher level (Supplementary Table 1). For example, the median F1-score of SCINA on Zheng sorted is 0.38, compared to a median F1-score of 1.0 for SCINA (using the original markers defined by the authors). However, SCINA only considers three cell populations: CD14+ monocytes, CD56+ NK cells, and CD19+ B cells. If we only consider these cell populations for SCINADE, this results in a median F1-score of 0.95.*

*We observed that the optimal number of marker-genes varies per classifier and dataset. For the Zheng sorted dataset the optimal number of markers is 5, 15, and 20 for DigitalCellSorterDE, GarnettDE, and, SCINADE respectively, while for Zheng 68K this is 5, 5, and 10. All together, these results illustrate the dependence of the classification performance on the careful selection of mark genes which is evidently a challenging task.”*

*• We added the following paragraph to the Methods section - Selecting marker-genes using differential expression:*

*“Using the cross-validation scheme, training data of each fold was used to select sets of 5, 10, 15, and 20 differentially expressed (DE) marker-genes. First, if the data was not already normalized, a CPM read count normalization was applied to the data. Next, the data was log-transformed using , and afterwards the DE test could be applied. As recommended in [27], MAST was used to find the DE genes [28]. The implementation of MAST in the FindAllMarkers() function of Seurat v2.3.0 was used to do a one-vs-all differential expression analysis [29]. Genes returned by Seurat were sorted and the top 5, 10, 15, or 20 significant genes with a positive fold change were selected as markergenes. These marker-genes were then used for population prediction of the test data of the corresponding fold. These marker-genes lists can be used by prior-knowledge classifiers such as SCINA, GarnettCV and DigitalCellSorter, by modifying the cell type marker-genes file required as an input to these classifiers. Such modification cannot be applied to the pre-trained classifiers of Garnettpretrained and Moana.”*

*• We added the following paragraph to the Results section - Performance evaluation across datasets:*

*“Second, we tested the classification performance on the PbmcBench datasets, which represent a more extensive protocol comparison. PbmcBench consists of two samples (pbmc1 and pbmc2), sequenced using seven different protocols (Table 2) with the exception that 10Xv3 was not applied to the pbmc2 sample. We used the pbmc1 datasets to evaluate the classification performance of all pairwise train-test combinations between the seven protocols (42 experiments, see Methods). Moreover, we extended the evaluation to include comparisons across different samples for the same protocol, using pbmc1 and pbmc2 (6 experiments, see Methods). All 48 experiments results are summarized in Figure 3. Overall, several classifiers performed well including SCINADE using 20 marker-genes, singleCellNet, scmapcell, scID and SVM, with an average median F1-score > 0.75 across all 48 experiments (Figure 3A, Supplementary Figure 9A). SCINADE, GarnettDE, and DigitalCellSorterDE were tested using 5, 10, 15 and 20 marker-genes, Figure 3A shows the best result for each classifier, where SCINADE and GarnettDE performed best using 20 and 5 marker-genes, respectively, while DigitalCellSorterDE had a median F1-score of zero during all experiments using all different numbers of marker-genes. DigitalCellSorterDE could only identify B-cells in the test sets, usually with an F1-score between 0.8 and 1.0, while the F1-score for all other cell populations was zero.”*

*• We modified the following paragraph in the Discussion section: “We evaluated the performance of the prior-knowledge methods (marker-based and pretrained) on PBMC datasets only, due to the limited availability of author-provided marker genes. For all PBMC datasets, the prior-knowledge methods did not improve the classification performance over supervised methods, which do not incorporate such prior knowledge. We extended some prior-knowledge methods such that the marker-genes were defined in a data-driven manner using differential expression which did not improve the performance of these classifiers, except for SCINADE (with 20 marker-genes) for the PbmcBench datasets. The data-driven selection of markers allows the prediction of more cell populations compared to the number of populations for which marker-genes were originally provided. However, this data-driven selection violates the fundamental assumption in prior-knowledge methods that incorporating expert-defined markers improves classification performance. Further, several supervised classifiers which do not require markers to be defined a priori (e.g. scPred and scID) already apply a differential expression test to find the best set of genes to use while training the model. The fact that prior-knowledge methods do not outperform other supervised methods and given the challenges associated with explicit marker definition, indicate that incorporating prior knowledge in the form of marker-genes is not beneficial, at least for PBMC data.”*

*• Updated Figure 1 with the differential expression results.*

*• Added Supplementary Figure 4.*

*• Added Figure 3.*

5. Apart from the Zheng et al datasets, all datasets used for comparison in this paper derive their underlying truth about the identity of each cell from the annotation of the original authors. This can obviously be biased. It would be great if the authors could benchmark the classifiers on an experimental mixture dataset, where the truth is known. One such dataset was recently published (Tian et al, Nature Methods 2019), it contains several datasets generated with different scRNA-seq protocols of experimental mixture of up to 5 cell lines.

*Response: We agree with the reviewer that it is important to benchmark the classifiers on datasets for which the ground truth is known, such as sorted populations or experimental mixture datasets as suggested. We have now included the suggested dataset in our benchmarking, which consists of two datasets representing a mixture of five lung cancer cell lines. The two datasets are obtained by applying two sequencing protocols, 10X and CEL-Seq2, to the same mixture. We included these datasets, now called CellBench, in the intra-dataset and the inter-dataset evaluations. Unfortunately, it turns out that the classification task for the CellBench datasets is quite easy, with almost all classifiers performing very well for both experimental setups (Figure 1 and Supplementary Figure 8).*

*We also added the Zheng sorted dataset to the evaluation, which consists of 10 FACS sorted pure PBMC populations. The Zheng dataset which was originally included, now called Zheng 68K to avoid confusion, is another PBMC dataset for which the cell labels were obtained by correlating the gene expression profile of each cell with the sorted populations of Zheng sorted and assign the cell to the population with the highest correlation. Thus, in some way the Zheng 68K is also clustered by the authors. The results show that some classifiers perform well on the sorted population, such as SVM, scPred and scVI (Figure 1). In general, we observed a lower performance across all classifiers when compared with other datasets, with the exception of Zheng 68K where the overlap between cell populations is quite high. The lower performance obtained on the Zheng sorted dataset compared to the other datasets can be explained by the fact that these sorted populations were defined based on the expression of specific protein markers, while the classification was done using transcriptomic data which is not always correlated with protein expression (van den Berg et al. 2017).*

*Changes to the manuscript:*

*• We added the following paragraph to the Results section - Benchmarking automatic cell*

*identification methods (intra-dataset evaluation): “For all previous datasets, cell populations were obtained through clustering. To assess how the classifiers perform when dealing with sorted populations, we included the CellBench dataset and the Zheng sorted dataset, representing sorted populations for lung cancer cell lines and PBMC, respectively.”*

*• We added the following paragraph to the Results section - All classifiers perform well in intra-dataset experiments:*

*“The CellBench 10X and CEL-Seq2 datasets represent an easy classification task, where the five sorted lung cancer cell lines are quite separable [14]. All classifiers have an almost perfect performance on both CellBench datasets (median F1-score ≈ 1).”*

*• We added the following paragraph to the Results section - Incorporating marker-genes does not improve intra-dataset performance on PBMC data:*

*“The classification of the Zheng sorted dataset is relatively easier compared to the Zheng 68K dataset, as almost all classifiers show improved performance (Figure 1), with the exception that LAmbDA failed while being tested on the Zheng sorted dataset. The prior-knowledge methods show high performance (median F1-score > 0.93), which is still comparable to other classifiers such as SVMrejection, scVI, scPred and SVM. Yet, the supervised classifiers do not require any marker-genes, and they can predict more (all) cell populations.”*

*• We added the following paragraph to the Results section - Performance evaluation across datasets:*

*“While evaluating the classification performance within a dataset (intra-dataset) is important, the realistic scenario in which a classifier is useful requires cross-dataset (i.e. inter-dataset) classification. We used 22 datasets (Table 2) to test the classifiers’ ability nto predict cell identities in a dataset that was not used for training. First, we tested the classifiers’ performance across different sequencing protocols, applied to the same samples within the same lab using the two CellBench datasets. We evaluated the classification performance when training on one protocol and testing on the other.*

*Similar to the intra-dataset evaluation result, all classifiers performed well in this case (Supplementary Figure 8).”*

*• We added the following paragraph to the Discussion section:*

*“The intra-dataset evaluation included the Zheng sorted dataset, which consists of 10 FACS sorted cell populations based on the expression of surface protein markers. Our results show relatively lower classification performance compared to other datasets, except the Zheng 68K dataset. The poor correlation between the expression levels of these protein markers and their coding genes mRNA levels [24] might explain this low performance.”*

*• We added the following paragraph to the Methods section - Inter-dataset classification: “CellBench. Both CellBench datasets, 10X and CEL-Seq2, were used once as training data and once as test data, to obtain predictions for the five lung cancer cell lines. The common set of detected genes by both datasets was used as features in this experiment.”*

*Updated Figure 1 to include results for the CellBench and Zhend sorted datasets.*

*• Added Supplementary Figure 8.*

6. It would be good to provide an overall ranking of the method in Figure 1. This way the reader can quickly see which method performs best overall.

*We updated all figures which compare performance (Figure 1, 3, 4, 5 and 6, Supplementary Figure 1 and 8) such that the classifiers (in rows) are ordered according to the mean performance of the experiments included in each figure, with the best classifier on top. Moreover, we added Figure 8 to the Discussion section summarizing the performance of all classifiers across all experiments carried out in the study.*

*Changes to the manuscript:*

*• Update ordering of Figures 1, 3, 4, 5 and 6, and Supplementary Figure 1 and 8.*

*• Added Figure 8 and Supplementary Table 4.*

Thank you for making your work reproducible.

**Reviewer 2**

Abdelaal et al. present a benchmarking study of single cell identification methods.

The study evaluates 20 available methods on multiple publicly available datasets using several quantitative metrics and conclude that SVM method is the best performing. The study looks

comprehensive and provide some insights. However, I have the following major concerns about the study:

1. There is no cross dataset validation on negative controls, i.e. when the cell types from one dataset should not be identified in the other dataset. In the scmap paper the SVM method was shown to perform poorly specifically in this case. I feel the main conclusion of the paper may be biased because of this.

*Response: To address the reviewer’s comment, we added two experiments to test the rejection option of the classifiers. The first experiment has the same setup used in the scmap paper (Kiselev et al. 2018) using negative controls. Briefly, we used two different mouse datasets, AMB16 (brain) and Baron Mouse (pancreas), and two different human datasets, Zheng 68K (PBMC) and Baron Human (pancreas). The second experiment mimics a more realistic scenario in which the test set contains a cell population (from the same tissue) that was not included in the training set.*

*For fairly assess the ability of SVM to reject unknown cells, we added an SVM with rejection option to the classifiers such that it could be tested in these experiments. SVM rejection was also added to all other experiments and turned out to be one of the top performing classifiers in most cases. Compared to SVM (without rejection), we observed that it always had a similar or higher median F1-score. We also noted that the percentage of unlabeled cells of SVM rejection is in most cases lower compared to other classifiers, such as scmapcell and scPred. We would like to note that while in the scmap paper, the authors used the R implementation of SVM from the e1071 package, we are using the python implementations of SVM from the scikitlearn package.*

*In the first experiment, we observed that scmapcluster and scPred had the best, almost perfect performance. SVM rejection and scmapcell performed almost perfect on all datasets except when being trained on Baron Mouse and being tested on AMB16 (Figure 6A).*

*In the second experiment, the performance of all classifiers decreased compared to the negative control experiment. The top performing classifiers during this experiment were scPred, SVM rejection, scID, scmapcell, and SCINA (Figure 6B).*

*Changes to the manuscript:*

*• We added the following paragraph to the Results section - Rejection option evaluation:*

*“Classifiers developed for scRNA-seq data often incorporate a rejection option to identify cell populations in the test set that were not seen during training. These populations cannot be predicted correctly and therefore should remain unassigned. To test whether the classifiers indeed leave these unseen populations unlabeled, we applied two different experiments using negative controls of different tissues and using unseen populations of the same tissue.*

*First, the classifiers were trained on a data set from one tissue (e.g. pancreas) and used to predict cell populations of a completely different tissue (e.g. brain) [20]. The methods should thus reject all (100%) of the cells in the test dataset. We carried out four different negative control experiments (see Methods, Figure 6A). scmapcluster and scPred have an almost perfect score for all four combinations, rejecting close 100% of the cells. Other top performing methods for this task, SVMrejection and scmapcell, failed when trained on mouse pancreatic data and tested on mouse brain data. All labeled cells of the AMB16 dataset are predicted to be beta cells in this case. The prior-knowledge classifiers, SCINA, Garnettpretrained, and DigitalCellSorter, could only be tested on the Baron Human pancreatic dataset. GarnettCV could, on top of that, also be trained on the Baron Human dataset and tested on the Zheng 68K dataset. During the training phase, GarnettCV tries to find representative cells for the cell populations described in the marker-genes file.*

*Being trained on Baron Human using the PBMC marker-genes file, it should not be able to find any representatives and therefore all cells in the Zheng 68K dataset should be unassigned. Surprisingly, GarnettCV still finds representatives for PBMC cells in the pancreatic data and thus the cells in the test set are labeled. However, being trained on the PBMC dataset and tested on the pancreatic dataset, it does have a perfect performance.*

*To test the rejection option in more realistic and challenging scenario, we trained the classifiers on some cell populations from one dataset, and used the held out cell populations in the test set (see Methods). Since the cell populations in the test set were not seen during training, they should remain unlabeled. Here, the difficulty of the task was gradually increased (Supplementary Table 3). First all the T-cells were removed from the training set. Next, only the CD4+ T cells were removed. Finally, only CD4+/CD45RO+ Memory T cells, a subpopulation of the CD4+ T cells, were removed.*

*The top performing methods for this task are: scmapcell, scPred, scID, SVMrejection, and SCINA (Figure 6B). We expected that rejecting T cells would be a relatively easy task as they are quite distinct from all other cell populations in the dataset. It should thus be comparable to the negative control experiment. Rejecting CD4+/CD45RO+ Memory T cells, on the other hand, would be more difficult as they could easily be confused with all other subpopulations of CD4+ T cells. Surprisingly, almost all classifiers, except for scID and scmapcluster, show the opposite.*

*To better understand this unexpected performance we analyzed the labels assigned by SVMrejection. In the first task (T Cells removed from the training set), SVMrejection labels almost all T cells as B cells. This can be explained by the fact that SVMrejection, and most classifiers for that matter, rely on classification posterior probabilities to assign labels but ignores the actual similarity between each cell and the assigned population. In task two (CD4+ T cells were removed), there were two subpopulations of CD8+ T cells in the training set. In that case, two cell populations are equally similar to the cells in the test set, resulting in low posterior probabilities for both classes and thus the cells in the test set remain unlabeled. If one of these CD8+ T cell populations was removed from the training set, only 10.53% instead of 75.57% of the CD4+ T cells were assigned as unlabeled by SVMrejection. All together, our results indicate that despite the importance of incorporating a rejection option in cell identity classifiers, the implementation of this rejection option remains challenging.”*

*• We added the following paragraph to the Methods section - Rejection:*

*“Negative control. Two human datasets, Zheng 68K and Baron Human, and two mouse datasets, AMB16 and Baron Mouse, were used. The Zheng 68K dataset was first stratified down sampled to 11% of its original size to reduce computation time. For each species, two different experiments were applied by using one dataset as training set and the other as test set and vice versa.*

*Unseen cell populations. Zheng 68K dataset was stratified downsampled to 11% of its original size to reduce computation time. Three different experiments were conducted.*

*First, all cell populations that are subpopulation of T cells were considered the test set. Next, the test set consisted of all subpopulations of CD4+ T cells. Last, only the CD4+/CD45RO+ Memory T cells were in the test set. Each time, all cell populations that were not in the test set, were part of the training set. Supplementary Table 3 gives an exact overview of the populations per training and test set.”*

*• Added Figure 6 and Supplementary Table 3.*

2. The cross dataset validation is only based on pancreatic datasets which have been extensively used in validation of the other cross datasets comparisons. The cell types (alpha, beta, delta and gamma) in the selected pancreas datasets are known to project very well to themselves. It would be interesting to look at other use cases of cross dataset validation using different tissues and cell types.

*We agree that the pancreatic datasets, especially using only the four major cell populations, are too easy to evaluate the performance of the classifiers accurately. Therefore, we added a recommendation to the discussion that developers should use more difficult datasets to test a new method.*

*In addition, we expanded our inter-dataset evaluations by adding three extra comparisons to enhance our results beyond the pancreatic datasets.*

*1. CellBench datasets - these datasets contain five sorted cell populations sequenced using two different protocols (10X and CEL-Seq2). We could thus use this to test the classifiers on sorted cell populations and to compare different protocols.*

*2. PbmcBench datasets - these datasets contain two different samples (pbmc1 and pbmc2) sequenced using seven different protocols. pbmc1 was used for pairwise comparison of all protocols and thus resulted in 42 extra experiments. Further, we compared the performance across different samples of the same protocol by training on pbmc1 and testing on pbmc2. This resulted in 6 extra experiments (for 10Xv3 pbmc2 was not sequenced). These experiments could thus be used for a more extensive protocol comparison.*

*3. Brain datasets - three datasets originating from different regions of the brain (VISp, ALM, and MTG) and different species (mouse, mouse, and human) and were sequenced using different technologies (single cell, single cell, and single nucleus), all from the same institute (Allen Institute for Brain Science). All datasets contained two levels of annotations: 3 and 34 cell populations. Here, all classifiers were tested on all possible train and test set combinations and on both annotation levels. This thus resulted in 18 extra experiments.*

*Changes to the manuscript:*

*• We added the following paragraph to the Results section - Performance evaluation across datasets:*

*“While evaluating the classification performance within a dataset (intra-dataset) is important, the realistic scenario in which a classifier is useful requires cross-dataset (i.e. inter-dataset) classification. We used 22 datasets (Table 2) to test the classifiers’ ability to predict cell identities in a dataset that was not used for training. First, we tested the classifiers’ performance across different sequencing protocols, applied to the same samples within the same lab using the two CellBench datasets. We evaluated the classification performance when training on one protocol and testing on the other.*

*Similar to the intra-dataset evaluation result, all classifiers performed well in this case (Supplementary Figure 8).*

*Second, we tested the classification performance on the PbmcBench datasets, which represent a more extensive protocol comparison. PbmcBench consists of two samples (pbmc1 and pbmc2), sequenced using seven different protocols (Table 2) with the exception that 10Xv3 was not applied to the pbmc2 sample. We used the pbmc1 datasets to evaluate the classification performance of all pairwise train-test combinations between the seven protocols (42 experiments, see Methods). Moreover, we extended the evaluation to include comparisons across different samples for the same protocol, using pbmc1 and pbmc2 (6 experiments, see Methods). All 48 experiments results are summarized in Figure 3. Overall, several classifiers performed well including SCINADE using 20 marker-genes, singleCellNet, scmapcell, scID and SVM, with an average median F1-score > 0.75 across all 48 experiments (Figure 3A, Supplementary Figure 9A). SCINADE, GarnettDE, and DigitalCellSorterDE were tested using 5, 10, 15 and 20 marker-genes, Figure 3A shows the best result for each classifier, where SCINADE and GarnettDE performed best using 20 and 5 marker-genes, respectively, while DigitalCellSorterDE had a median F1-score of zero during all experiments using all different numbers of marker-genes. DigitalCellSorterDE could only identify B-cells in the test sets, usually with an F1-score between 0.8 and 1.0, while the F1-score for all other cell populations was zero.*

*We also tested the prior-knowledge classifiers on all 13 PbmcBench datasets. The priorknowledge classifiers showed lower performance compared to other classifiers (average median F1-score < 0.6), with the exception of SCINA which was only tested on three cell populations (Figure 3B, Supplementary Figure 9B). These results are in line with our previous conclusions from the Zheng sorted and Zheng 68K datasets in the intra-dataset evaluation.*

*Comparing the performance of the classifiers across the different protocols, we observed a higher performance for all classifiers for specific pairs of protocols. For example, all classifiers performed well when trained on 10Xv2 and tested on 10Xv3, and vice versa.*

*On the other hand, other pairs of protocols had good performance only in one direction, training on Seq-Well produced good predictions on 10Xv3, but not the other way around.*

*Compared to all other protocols, the performance of all classifiers was low when they were either trained or tested on Smart-seq2 data. This can, in part, be due to the fact that Smart-seq2 data does not contain Unique Molecular Identifier (UMI), in contrast to all other protocols.*

*We also tested the classification performance using the three brain datasets, VISp, ALM and MTG (Table 2), which allowed us to compare performances across species (mouse and human) as well as single-cell RNA-seq (used in VISp and ALM) versus singlenucleus RNA-seq (used for MTG). We tested all possible train-test combinations for both levels of annotation, three major brain cell types (inhibitory neurons, excitatory neurons and non-neuronal cells) and the deeper annotation level with 34 cell populations (18 experiments, see Methods). Prediction of the three major cell types was easy, where almost all classifiers showed high performance (Figure 4A) with some exceptions. For example, scPred failed the classification task completely when testing on the MTG dataset, producing 100% unlabeled cells (Supplementary Figure 10A). Predicting the 34 cell populations turned out to be a more challenging task, especially when the MTG human dataset is included either as training or testing data, resulting in significantly lower performance across all classifiers (Figure 4B). Across all nine experiments at the deeper annotation, the top performing classifiers were SVM, ACTINN, singleCellNet, SingleR and LAmbDA, with almost 0% unlabeled cells (Supplementary Figure 10B).”*

*• We modified the following paragraph in the Discussion:*

*“Overall, we observed that the performance of almost all methods was relatively high on various datasets, while some datasets with overlapping populations (e.g. Zheng 68K dataset) remain challenging. The inter-dataset comparison requires extensive development in order to deal with technical differences between protocols, batches, and labs, as well as proper matching between different cell population annotations. Further, the pancreatic datasets are known to project very well across studies and hence using them to evaluate inter-dataset performance can be misleading. We recommend considering other challenging tissues and cell populations.”*

*• We added the following paragraph to the Methods section - Inter-dataset classification: “CellBench. Both CellBench datasets, 10X and CEL-Seq2, were used once as training data and once as test data, to obtain predictions for the five lung cancer cell lines. The common set of detected genes by both datasets was used as features in this experiment.*

*PbmcBench. Using pbmc1 sample only, we tested all train-test pairwise combinations between all seven protocols, resulting in 42 experiments. Using both pbmc1 and pbmc2 samples, for the same protocol we used pbmc1 as training data and pbmc2 as test data, resulting in six additional experiments (10Xv3 was not applied for pbmc2). As we are now dealing with PBMC data, we evaluated all classifiers, including the prior-knowledge classifiers, as well as the modified versions of SCINA, GarnettCV and DigitalCellSorter, in which the marker-genes are obtained through differential expression from the training data as previously described. Through all these 48 experiments, genes that are not expressed in the training data were excluded from the feature space. Also, as these PbmcBench datasets differ in the number of cell populations (Table 2), only cell populations provided by the training data were used for the test data prediction evaluation.*

*Brain. We used the three brain datasets, VISp, ALM and MTG with two levels of annotations, 3 and 34 cell populations. We tested all possible train-test combinations, by either using one dataset to train and test on another (6 experiments) or using two concatenated datasets to train and test on the third (3 experiments). A total of nine experiments was applied for each annotation level. We used the common set of detected genes between the datasets involved in each experiment as features.”*

*• Added Figure 3 and 4, and Supplementary Figure 8, 9, and 10.*

3. I feel that with the current growth of publicly available datasets (various mouse atlases) the selection of datasets for the study could be significantly extended. At the moment the datasets used in the study have been very well studied and described before and may not be the best for providing new useful insights and guidance.

*Response: Although we agree with the reviewer that the current growth and availability of scRNA-seq datasets allows for more comparisons, we also believe that adding more datasets should be well motivated to avoid drowning the readers in the results.*

*That being said, in the revised version of the manuscript we have added 19 new datasets, all published in 2018 and 2019 and not used in the original publications of the classifiers, except the Zheng sorted dataset.*

*• The two CellBench datasets represent a mixture of five lung cancer cell lines sequenced using two different protocols, 10X and CEL-Seq2.*

*• The Zheng sorted dataset consists of 10 purely FACS sorted PBMC populations. We added these datasets as a different type of datasets for which the true labels are known and not obtained through clustering by the authors.*

*• The CellBench datasets are used for both inter- and intra-dataset experiments, while the Zheng sorted dataset is only used for intra-dataset evaluation. The 13 PbmcBench datasets composed of two pbmc samples measured using seven different protocols. The PbmcBench datasets were used to compare different protocols using the same sample, and same protocol across different samples.*

*• The three brain datasets, VISp, ALM and MTG, from the Allen Institute, representing three brain regions. Two datasets are mouse, and one is human. These datasets represent comparison across species (mouse and human), and also single-cell RNA-seq (used in VISp and ALM) versus single-nucleus RNA-seq (used for MTG).*

*In total, our benchmarking study now includes 27 datasets which we believe allows a comprehensive evaluation of the different aspects of automatic cell identification. We would like to reiterate that in addition to making all our code available to reproduce our results, we also provide a snakemake workflow which makes it easy to compare all the classiers on any other dataset.*
